# Treatment of humerus fractures in the elderly: A systematic review covering effectiveness, safety, economic aspects and evolution of practice

**DOI:** 10.1371/journal.pone.0207815

**Published:** 2018-12-13

**Authors:** Cecilia Mellstrand Navarro, Agneta Brolund, Carl Ekholm, Emelie Heintz, Emin Hoxha Ekström, Per Olof Josefsson, Lina Leander, Peter Nordström, Lena Zidén, Karin Stenström

**Affiliations:** 1 Department of Hand Surgery, Karolinska Institute, Institution for Clinical Research and Education, Södersjukhuset Hospital, Stockholm, Sweden; 2 Swedish Agency for Health Technology Assessment and Assessment of Social Services, Stockholm, Sweden; 3 Department of Orthopaedics, Sahlgrenska University Hospital, Gothenburg, Mölndal, Sweden; 4 Department of Learning, Informatics, Management and Ethics (LIME), Karolinska Institutet, Stockholm, Sweden; 5 Department of Orthopaedics, Skane University Hospital, Malmö, Sweden; 6 Department of Community Medicine and Rehabilitation, Geriatrics, Umeå, Sweden; 7 Department of Health and Rehabilitation, The Sahlgrenska Academy at the University of Gothenburg, Institute of Neuroscience and Physiology, Gothenburg, Sweden; Consorci Parc de Salut MAR de Barcelona, SPAIN

## Abstract

**Objectives:**

The objective of this Health Technology Assessment was to evaluate effectiveness, complications and cost-effectiveness of surgical or non-surgical treatment for proximal, diaphyseal or distal fractures of the humerus in elderly patients. Secondary objectives were to evaluate the intervention costs per treatment of proximal humerus fractures (PHF) and to investigate treatment traditions of PHF in Sweden.

**Methods and findings:**

The assessment contains a systematic review of clinical and health economic studies comparing treatment options for humerus fractures in elderly patients. The results regarding the effectiveness of treatments are summarized in meta-analyses. The assessment also includes a cost analysis for treatment options and an analysis of registry data of PHF. For hemiarthroplasty (HA) and non-operative treatment, there was no clinically important difference for moderately displaced PHF at one-year follow-up regarding patient rated outcomes, (standardized mean difference [SMD]) -0.17 (95% CI: -0.56; 0.23). The intervention cost for HA was at least USD 5500 higher than non-surgical treatment. The trend in Sweden is that surgical treatment of PHF is increasing. When functional outcome of percutaneous fixation/plate fixation/prosthesis surgery and non-surgical treatment was compared for PHF there were no clinically relevant differences, SMD -0.05 (95% CI: -0.26; 0.15). There was not enough data for interpretation of quality of life or complications. Evidence was scarce regarding comparisons of different surgical options for humerus fracture treatment. The cost of plate fixation of a PHF was at least USD 3900 higher than non-surgical treatment, costs for complications excluded. In Sweden the incidence of plate fixation of PHF increased between 2005 and 2011.

**Conclusions:**

There is moderate/low certainty of evidence that surgical treatment of moderately displaced PHF in elderly patients has not been proven to be superior to less costly non-surgical treatment options. Further research of humerus fractures is likely to have an important impact.

## Introduction

Treatment of humerus fractures in the elderly remains a therapeutic challenge. These injuries are among the most common fractures [1] and cause large expenses for the individual and for society, due to their high frequency, the surgical complexity, and risk of poor outcome. There are indications that the incidence of osteoporosis related proximal humerus fractures (PHF) is increasing [2, 3], with an incidence of 10,5/10,000 in people aged 60 or older in a Scandinavian population [3].

Although non-surgical treatment is a reasonable treatment option for the majority of humerus fractures [4–6], there is an increasing interest in surgical intervention [7, 8]. New technical possibilities for fracture fixation in the elderly were evident after the introduction of angle stable implants at the turn of the 21^st^ century [9]. The use of shoulder arthroplasties as fracture treatment has undergone a rapid development during the 21^st^ century [10]. Only few comparing studies have been performed investigating effectiveness and complications after fracture surgery, and the need for prospective randomized trials has been advocated [11]. New surgical methods for humerus fracture care have been introduced and widespread before they have been scientifically evaluated [10]. There are no evidence-based treatment recommendations, thus permitting large local variation in treatment preferences [7, 10–12]. Moreover, societal costs are increasing for osteoporosis fracture health care [13], and there is reason to believe that more sophisticated surgical methods for treatment of humerus fractures will add more expenses in the future. However, to the best of our knowledge, health economic assessments of humerus fractures are largely lacking.

Heath Technology Assessment (HTA) is a scientific methodology used to gather and summarize scientific data to influence policy and clinical decision making on the use of health technologies. In this context, the primary aim of this HTA analysis was to assess the literature describing the effectiveness, complications and the cost-effectiveness of treatment options for humerus fractures in a population with a mean age above 60 years. Other aims were to evaluate the intervention costs for treatments of proximal humerus fractures and to investigate the Swedish national incidence rates of PHF and their treatment during the period 2005–2013.

## Materials and methods

The present HTA analysis includes a systematic literature review of clinical and health economic studies comparing treatment options for fractures of any part of the humerus. The results regarding the effectiveness of the treatments are summarized in meta-analyses. In addition, the assessment contains a cost analysis for different treatment options commonly used for PHF care. Lastly, this HTA report includes an analysis of registry data providing information on the incidence and treatment traditions concerning PHF during the period 2005–2013. The present work was conducted and funded within the framework for the Swedish Agency for Health Technology Assessment and Assessment of Social Services, SBU (http://www.sbu.se/en), a Swedish public agency conducting health technology assessments. The process during which this HTA analysis was performed, has continuously been reviewed by an internal group for quality control of the Swedish Agency for Health Technology Assessment and Assessment of Social Services. After completion, its accuracy was audited by an external council of medical experts.

### Systematic review and meta-analysis

#### Protocol and registration

The systematic literature review was based upon studies investigating benefits and possible risks of different methods for treating humeral fractures in a study population with mean age of at least 60 years. Methods of analysis and inclusion criteria for the project were specified in advance, as a part of the internal process at SBU. No protocol has been published.

#### Eligibility criteria

The criteria for eligibility were outlined according to the PICOS model (Population, Intervention, Comparator, Outcome and Study design) and included the following characteristics:

**Table:** 

**Population**	The mean age of the study population 60 years or above. All study participants were treated for a fracture of any part of the humerus. Studies on cadavers were excluded.
**Interventions**	Any operative or non-operative fracture treatment.
**Comparator**	Any comparator (e.g., any alternative treatment, operative or non-operative).
**Outcome and measures**	Functional outcomes, adverse effects/complications, quality of life (QoL), cost-effectiveness and costs. Any validated measure was acceptable.
**Study design**	Randomized controlled trial (RCT), non-randomized controlled (Non-R) studies and comparative registry studies.
**Setting**	Any setting.
**Language**	Studies published in English or in the Scandinavian languages.
**Publication type**	Studies published in peer-reviewed journals.

**Information sources**. Studies were identified by searching electronic databases and by scanning the reference lists of studies meeting the eligibility criteria, and of relevant systematic reviews. The electronic databases PubMed, EMBASE, Cochrane Library, and Scopus were searched from January 1990 to December 2016.

#### Search strategy

Electronic searches were conducted using a combination of medical subject headings (MeSH) and relevant text word terms related to fractures of the upper extremity, old age, interventions and study type. (For detailed information about the search strategies, see S1 Appendix.)

#### Study selection

Six reviewers, all expert senior scientists, independently screened the titles and abstracts for eligibility. Each abstract was screened by two reviewers. The abstracts were screened and rated using the online available scanning tool Rayyan [14]. All publications of potential relevance according to the inclusion criteria were retrieved in full text. Eligibility for inclusion was independently assessed by two reviewers. Disagreements were resolved by consensus. Reference lists of studies meeting the eligibility criteria and of relevant systematic reviews were screened for additional relevant studies. (For detailed information about the included studies, see S2 Appendix, excluded studies, see S3 Appendix.)

#### Risk of bias in individual studies

To determine the internal validity of the eligible trials, a pair of reviewers independently assessed the risk of bias according to the SBU checklist [15]. The SBU checklist for the inclusion of studies, which has been described in detail previously [16] “is based on the CONSORT statement and discloses risk of bias related to six main aspects: selection; treatment (including blinding); measurement; attrition; reporting; and conflicts of interest [17]. The checklist was used to reveal shortcomings of the studies. The reviewers thereafter assessed the extent to which the internal validity of the results could have been affected by these shortcomings. A rating of low, moderate or high risk of bias was given to each category of items. Based on the severity of the combined threats to internal validity, an overall rating of risk of bias was then given to each study.” For the health economic studies, a specific check-list for within trial cost-effectiveness studies was used [18]. Only studies with a low or moderate overall risk of bias were included in the synthesis.

#### Data items

The following information was extracted from the included trials: (1) Type of injury, study design, time to follow-up, period when the study was performed (yrs.); (2) Number of participants, mean age and sex; (3) treatment, drop-out rate, side-effects; (4) type of comparator, drop-out rate, side-effects (5) outcome and measures; (6) risk of bias.

#### Data collection process

Data was extracted from each included study and inserted into a table by one reviewer. A second reviewer audited the data extraction. Any disagreements were resolved by discussion. Functional outcome was reported by validated assessment instruments, e.g. Disabilities of the Arm, Shoulder, and Hand (DASH) [19] and Constant and Murley Score (Constant score) [20]. Quality of life was presented if reported by validated quality of life instruments, such as EuroQoL 5 Dimensions (EQ-5D) [21, 22] and 15-Dimensional (15-D) [23]. Minimal clinically important differences (MCID) scores for different outcomes (Table 1) were used to reflect the clinical importance of the measured differences between the treatments. Complications were defined as major if they demanded surgical treatment or produced permanent serious disability. All other complications were defined as minor. Each complication was presumed to occur in one individual, even if some patients inevitably may have been affected by two or more complications, thus overestimating the number of patients with complications. Any statistically significant difference in complications was considered clinically important.

**Table 1:** Outcome measurements used in this HTA analysis regarding fractures of the proximal humerus, with corresponding minimal clinically important differences (MCID).

Outcomes	MCID	References
Constant score	10 Points	[24, 25]
EQ-5D	0.074 Points	[26, 27]
DASH	13 points	[28–31]

#### Statistical methods

The software Comprehensive Meta-Analysis (CMA), Version 3.3, software (Biostat NJ, USA) was used for the meta-analyses. Random effect models were applied, due to the substantial heterogeneity that was expected regarding populations, interventions, comparators and outcome measures across studies. The principal summary measures were mean difference (MD) with 95% CI for the final follow-up assessment. The summary measure standardized mean difference (SMD, Cohen’s d), based on the groups’ sample sizes, means and standard deviations were used when summaries of measures were not possible to be presented as MD. In case of only one RCT forming the results, Non-R studies and registry studies were also used to illustrate the differences between treatment options. For analysis of complications, meta-analysis was performed through calculation of Risk Difference (RD), presented as percentages. All results are presented for one year follow up, or longer follow-up times when data was available. Inconsistencies and heterogeneity disclosed by the meta-analyses were considered when the certainty of evidence across studies was assessed.

#### Assessing certainty of evidence across studies using GRADE

The international system GRADE [32] was used to assess the certainty of evidence for efficacy, effectiveness and complications across studies according to the following four levels, as described in detail previously [33]:

High–We are very confident that the true effect lies close to that of the estimate of the effect. This level is illustrated in Tables 2, 3, and 4 (⊕⊕⊕⊕).

**Table 2:** Certainty of evidence for surgical vs. non-surgical treatment options of proximal humerus fractures in the elderly. N/A = Not Applicable.

Intervention	Outcome measure	Comparator (C)	N_RCT+Cohort_/Trials [Reference(s)]	Results	Certainty of evidence (GRADE)	Comment
**Surgical vs. non-surgical methods—proximal humerus fractures**						
Hemiarthroplasty (HA)	Function	Sling	101/ 2 RCTs/[52, 56]	No clinically important difference	(⊕⊕❍❍)	-2 indirectness
				SMD -0.17 (-0.56; 0.23)		
Hemiarthroplasty (HA)	Quality of life	Sling	54/1 RCTs/[52]	N/A	(⊕❍❍❍)	Single study
Hemiarthroplasty (HA)	Complications- minor	Sling	No study	N/A- minor	(⊕❍❍❍)	
Hemiarthroplasty (HA)	Complications- major	Sling	101/ 2 RCTs/[52, 56]	N/A- major	(⊕❍❍❍)	-1 risk of bias
						-2 indirectness
Different types of internal fixation	Function	Sling	373+231/4 RCTs and 3 cohorts/[50, 51, 55, 57–60]	No clinically important difference)	(⊕⊕⊕❍)	-1 indirectness
				SMD -0.05 (-0.26; 0.15		
Different types of internal fixation	Quality of life	Sling	277/3 RCTs/[50, 51, 55]	No clinically important difference	(⊕⊕❍❍)	-1 risk of bias
				MD -0.01 (-0.06; 0.05)		-1 indirectness
Different types of internal fixation	Complications-minor	Sling	44+129/2 RCTs and 1 cohort/[55, 57, 58]	N/A	(⊕⊕❍❍)	-1 risk of bias
						-2 indirectness
Different types of internal fixation	Complications -major	Sling	373+129/ 4 RCTs and 1 cohort/[50, 51, 55, 57, 58]	No clinically important difference regarding *major* complications	(⊕⊕❍❍)	-1 risk of bias
				RD 0.07 (-0.06; 0,20)		-1 inconsistency

**Table 3:** Certainty of evidence for surgical treatment options of proximal humerus fractures in the elderly. N/A = Not Applicable.

Intervention	Outcome (measure)	Comparator (C)	N_RCT+Cohort_/Trials [Reference(s)]	Results	Certainty of evidence (GRADE)	Comment
Surgical vs. surgical methods—proximal humerus fractures						
Plates with monoaxial locking screws	Function	Plates with polyaxial locking screws	56+76/1 RCT and 1 cohorts/ [65, 66]	-	(⊕❍❍❍)	N/A
Plates with monoaxial locking screws	Quality of life	Plates with polyaxial locking screws	**-**	-	(⊕❍❍❍)	No study
Plates with monoaxial locking screws	Complications	Plates with polyaxial locking screws	56+76/1 RCT and 1 cohorts/ [65, 66]	-	(⊕❍❍❍)	N/A

Plates with enhanced support	Function	Plates without enhanced support	122/ 2 RCTs/ [53, 67]	-	(⊕❍❍❍)	N/A
Plates with enhanced support	Quality of life	Plates without enhanced support	**-**	-	(⊕❍❍❍)	No study
Plates with enhanced support	Complications	Plates without enhanced support	122/ 2 RCTs + 1 cohort/ [53, 67, 68]	-	(⊕❍❍❍)	N/A

Different kinds of internal fixation	Function	Prosthesis	60+104 /1 RCT and 2 cohorts/[54, 69, 70]	-	(⊕❍❍❍)	
Different kinds of internal fixation	Quality of life	Prosthesis	44/ 1 cohort/[69]	-	(⊕❍❍❍)	
Different kinds of internal fixation	Complications	Prosthesis	60+104 /1 RCT and 2 cohorts/[54, 69, 70]	-	(⊕❍❍❍)	

Plate fixation	Function	Intramedullary nail	65+193/1 RCT and 2 cohorts/[71–73]	No clinically important difference MD_RCT_, -3.8 (95% CI: -11.85 to 4.25) MD_cohorts_: 4.51 (95% CI: -0.99 to 10.1)	(⊕⊕❍❍)	Single study
Plate fixation	Quality of life	Intramedullary nail	41/1 cohort/[73]	**-**	(⊕❍❍❍)	Single study
Plate fixation	Complications	Intramedullary nail	65+328/1 RCT and 3 cohorts/[71–74]	-	(⊕❍❍❍)	-1 indirectness
						-2 inconsistency

Reverse shoulder arthroplasty (RSA)	Function	Hemiarthroplasty (HA)	61+448/1 RCT and 3 cohort/[61–64]	Statistically significant difference RSA>HA	(⊕⊕❍❍)	-1 risk of bias
				MD_RCT_ 6.9 (95% CI: 3.0 to 10.8)		-1 indirectness
Reverse shoulder arthroplasty (RSA)	Quality of life	Hemiarthroplasty (HA)	-	-	(⊕❍❍❍)	No study
Reverse shoulder arthroplasty (RSA)	ComplicationsMinor and major	Hemiarthroplasty (HA)	Minor: 62+80/1 RCT and 2 cohorts /[61–63] Major: 368/ 1 cohort/[64]	-	(⊕❍❍❍)	-2 indirectness-1 inconsistency

Different kinds of fixation of the tubercles during arthroplasty	Function	Different kinds of fixation of the tubercles during arthroplasty	35+54/1 RCT and 1 cohort/[75, 76]	-	(⊕❍❍❍)	-1 risk of bias
						-1 indirectness -1 inconsistency
Different kinds of fixation of the tubercles during arthroplasty	Quality of life	Different kinds of fixation of the tubercles during arthroplasty	-	-	(⊕❍❍❍)	No study
Different kinds of fixation of the tubercles during arthroplasty	Complications	Different kinds of fixation of the tubercles during arthroplasty	35/1 RCT/[75]	-	(⊕❍❍❍)	Single study

Straight intramedullary nail	Function	Curved intramedullary nail	52/1 RCT/[77]	-	(⊕❍❍❍)	Single study
Straight intramedullary nail	Quality of life	Curved intramedullary nail	-	-	(⊕❍❍❍)	No study
Straight intramedullary nail	Complications	Curved intramedullary nail	52/1 RCT/[77]	-	(⊕❍❍❍)	Single study

**Table 4:** Certainty of evidence for comparisons of non-surgical treatment options of proximal humerus fractures in the elderly.

Intervention	Outcome measure	Comparator (C)	N_RCT+Cohort_/Trials [Reference(s)]	Results	Certainty of evidence (GRADE)	Comment
**Surgical vs. surgical methods- proximal humerus fractures**						
Early mobilization after hemiarthroplasty (HA)	Function	Late mobilization after hemiarthroplasty (HA)	49/1 RCT/ [78]	-	(⊕❍❍❍)	One study
Early mobilization after hemiarthroplasty (HA)	Quality of life	Late mobilization after hemiarthroplasty (HA)		-	(⊕❍❍❍)	No study
Early mobilization after hemiarthroplasty (HA)	Complications	Late mobilization after hemiarthroplasty (HA)	49/1 RCT/ [78]	-	(⊕❍❍❍)	One study

Moderate–We are moderately confident in the effect estimate: The true effect is likely to be close to the estimate of the effect, but there is a possibility that it is substantially different. This level is illustrated in Tables 2, 3 and 4 (⊕⊕⊕❍).

Low–Our confidence in the effect estimate is limited: The true effect may be substantially different from the estimate of the effect. This level is illustrated in Tables 2, 3 and 4 (⊕⊕❍❍).

Very low–We have very little confidence in the effect estimate: The true effect is likely to be substantially different from the estimate of the effect. This level is illustrated in Tables 2, 3 and 4 (⊕❍❍❍).

The GRADE system postulates that scientific evidence based on data from RCTs is assessed as high certainty. The level of confidence for either study type may then be downgraded due to several circumstances noted in each particular study, such as risk of bias, inconsistency, indirectness, imprecision and publication bias. There was no upgrading in confidence based on large magnitude of effect or dose response. The rating of certainty of evidence in our literature review was decided through consensus among the authors supported by GRADE guidelines.

### Cost analysis

Direct costs of the primary treatment opportunity in primary or inpatient care for the different treatments for PHF care in a Swedish setting were estimated using a bottom up approach [34]. In this approach, the resources necessary to provide each treatment are first identified and then valued using unit costs for each unit of resources. Four different operative treatments as well as a non-operative alternative were included in the analysis: Plate fixation, Intramedullary (IM) nail fixation, Hemiarthroplasty (HA), Reverse shoulder arthroplasty (RSA)) and non-operative treatment with a sling.

The cost analysis included the cost for staff, operating theatre rental costs (including overhead costs), costs for medical technical equipment, costs for orthopedic implants, and costs for consumables. For a detailed description, see S5 Appendix. Calculations were performed only for costs related to the primary treatment opportunity in inpatient care, i.e. no calculations were performed regarding for example out-patient clinic follow-ups, or societal costs. Short- or long-term complications were not included in the analysis due to lack of published data. All resources needed for each treatment method were identified by expert senior scientists in the research group. Time in operating theatre for each type of surgical intervention was estimated from previous publications [35–38]. Time consumption for non-surgical interventions, pre- and post-operative care, hospital inpatient duration and utilization of staff involved in different stages of the different treatments, were estimated by expert senior scientists in the research group based on clinical experience and reported data from three large Swedish general hospitals’ computerized operation planning systems. The time for pre-and post-operative care in the perioperative intensive care ward was estimated to be equally long for all surgical treatments, see detailed description in S5 Appendix.

The material costs (consumables and implants) were estimated by collecting data from the economic departments of three general hospitals in Sweden (The Sahlgrenska University hospital, Gothenburg, Skåne University hospital, Malmö, and the hospital Södersjukhuset, Stockholm). The cost for material was valued using a conservative approach by always using the product with the lowest procured price. The units and unit costs derived from these sources are presented in S5 Appendix. All costs were converted to US dollars in 2016 using the method recommended by The Cochrane and Campbell Economic Methods Group, i.e. with PPPs (purchasing power parity) via The CCEMG–EPPI-Centre Cost Converter (v.1.5 last update: 29 April 2016) http://eppi.ioe.ac.uk/costconversion/ (IMF-PPP).

### Registry analysis

All Swedish health-care providers supply mandatory registration of all out- and in-patient care given in Sweden. Registered data is collected in the Swedish National Patient Registry kept by the National Board of Health and Welfare (https://www.socialstyrelsen.se/english). For the purpose of the present study, in- and outpatient clinic data was ordered for the period 2005–2013 with a selection of all individuals above the age of 50 with a reported International Classification of Disease edition 10 (ICD-10) code [39] for a PHF. Individuals were counted only once to avoid overestimation of incidences. If a Nordic code for surgical procedures (NOMESCO) code [40] for surgical intervention of PHF occurred in the registry within 30 days of the date of a PHF code, the fracture was considered to have been treated surgically. All other fractures were considered to have been treated non-operatively. Incidences were calculated as the number of events divided by the size of the target population according to Statistics Sweden (http://www.scb.se/en/). The data was connected to the Swedish Causes of Death Registry (http://www.socialstyrelsen.se/register/dodsorsaksregistret) to calculate the risk of death after proximal humerus injury. All handling of data and calculations of registry data were performed in SPSS (version 23; IBM, NY, USA).

### Ethics

No ethical permit was collected for this literature review. All patient data was collected from published data. All registry data used in this study was unidentified and approved by the National Board of Health and Welfare in Sweden.

## Results

### Systematic review and meta-analysis

#### Eligible studies

**Fig 1:**
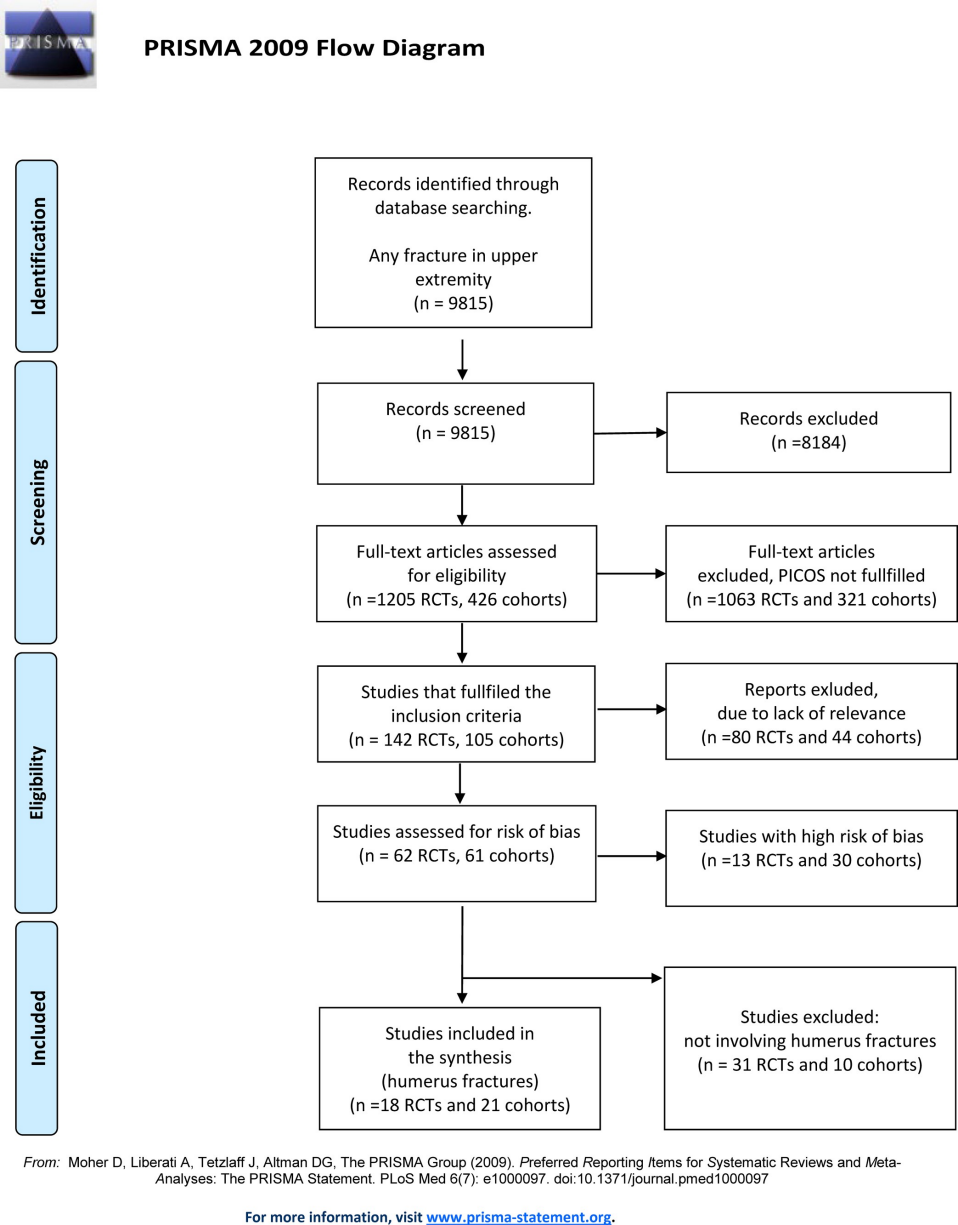
PRISMA flow-chart.

The literature search of studies investigating the effectiveness of treatments for the upper extremity yielded a total of 9815 citations: after review of the abstracts, 8184 were discarded. The full text of a total of 1205 RCTs and 426 cohort studies citations was examined. 1063 RCTs and 321 cohort studies were excluded since the studies did not meet the inclusion criteria, thus leaving 142 RCTs and 105 cohorts to be evaluated for relevance. Out of these, 80 RCTs and 44 cohort studies were deemed as not relevant, leaving 62 relevant RCTs and 61 relevant cohort studies. After the assessment of risk of bias, 13 RCTs and 30 cohorts were considered to be of high risk of bias, leaving 49 RCTs and 31 cohort studies with low/moderate risk of bias (see S2 Appendix for included studies and S3 Appendix for excluded studies). Studies investigating the forearm (radius and ulna) were finally discarded, leaving 18 RCTs and 21 Non-R studies regarding humerus fractures for analysis (Fig 1).

The search for health economic studies regarding fracture treatment in the upper extremity yielded a total of 569 potentially relevant citations. Out of these, 118 were read in full text and nine of these were judged to be relevant studies of treatments for humerus fractures. Six of these studies focused only on resource use or costs [41–46] and only three remaining studies were full cost-utility analyses (CUA) [47–49] (see S2 Appendix for included studies and S3 Appendix for excluded studies).

#### Risk of bias in individual studies

All included studies were deemed to have moderate or low risk of bias based on the methodological assessment performed by authors CE, CMN, LZ, PN, POJ and LEO (the latter mentioned in the Acknowledgement section). Included studies and their risk of bias are presented in S6 Appendix. All studies presented data with a mean age of study participants that cohered to the inclusion criteria of this meta-analysis. However, some studies included only elderly patients whereas other studies included all ages but with a mean age of participants that met the inclusion criteria for this meta-analysis, S6 Appendix. Differences in age distribution among studies may have introduced bias. Fracture severity was not always described in full detail and represents a risk of bias. Most studies regarding proximal humerus fractures presented included fractures by the Neer classification as listed in S6 Appendix. Exclusion criteria however, differed between studies and may introduce a risk of bias. Olerud and Handoll for example [50–52], excluded patients due to indication for mandatory surgical treatment. This definition however, was not explained in full detail. Only Chen and Liu [53, 54] defined the level of mineral bone density as an inclusion criterion, a detail which maybe would have been appropriate for all patient populations to avoid bias. Most results were collected from the 1-year follow-up, but some results were collected from a later follow-up point (S6 Appendix) which may represent a risk of bias. In studies comparing surgery and non-operative treatment, blinding was not possible to achieve. No study described a double-blind allocation and assessment.

### Comparison of non-surgical and surgical treatment–Proximal humerus fractures

#### Study characteristics

Nine trials, six RCTs (474 participants) and three Non-R studies (231 participants) were included that compared non-surgical and surgical treatment of PHF in the elderly. The subjects were predominantly female. All nine studies were conducted in Europe. The treatment methods under study were HA or plate fixation compared with non-surgical treatment (sling: body bandage). No study included fractures demanding imperative surgical treatment.

#### Hemiarthroplasty (HA) vs. non-surgical treatment

**Fig 2:**
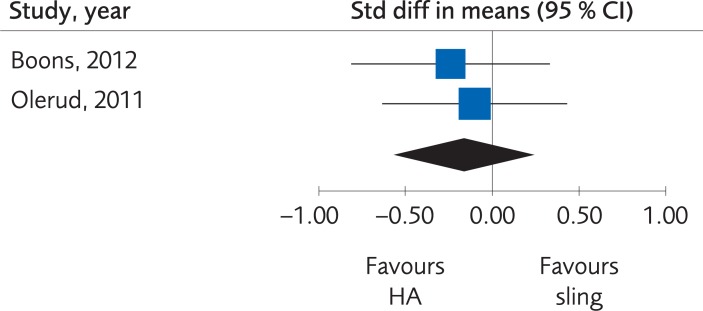
Meta-analysis of randomized controlled trials comparing hemiarthroplasty (HA) with sling immobilization: results of functional outcome, in elderly patients with proximal humerus fractures at one-year follow-up.

*Results*. Two RCTs compared HA with sling immobilization regarding the functional outcome results as measured by Disabilities of the Arm, Shoulder and Hand (DASH) Score and Constant score. There was no clinically important difference, at one-year follow-up, SMD -0.17; 95% CI: -0.56 to 0.23 (Fig 2). Due to the low number of trials, the certainty of evidence was rated as low.

#### Operative vs. non-operative treatment

**Fig 3:**
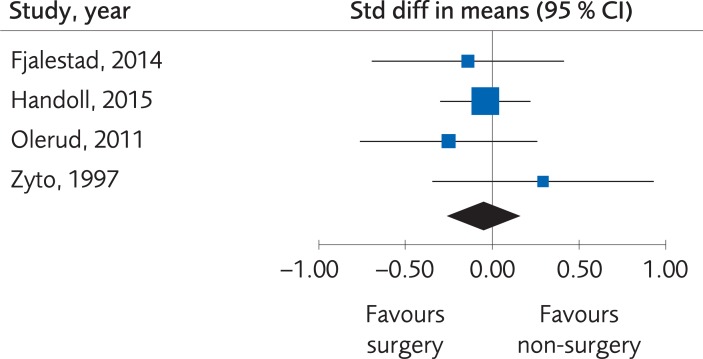
Meta-analysis of randomized controlled trials comparing different methods for internal fixation with treatment with a sling: results of functional outcome, in elderly patients with proximal humerus fractures at one-year follow-up.

**Fig 4:**
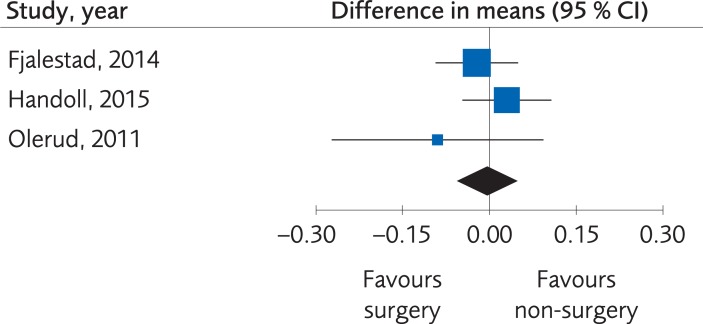
Meta-analysis of randomized controlled trials comparing different methods for internal fixation with treatment with a sling: results of quality of life, in elderly patients with proximal humerus fractures at one-year follow-up.

*Results*. Four RCTs (373 subjects) and three Non-R studies (231 subjects) compared functional outcomes (DASH Score, Constant score or Oxford shoulder score (OSS)) between different methods for surgical intervention (percutaneous fixation, plate fixation, nail or arthroplasty) and sling immobilization. There were no clinically important differences regarding the functional outcome, SMD -0.05; 95% CI: -0.26 to 0.15, Fig 3, at one-year follow-up, and the evidence was rated as being of moderate quality due to the limited number of participants. Quality of life was measured in three of these studies, using the EQ-5D in two [50, 51] and 15-D in the one by Fjalestad et al., 2014 [55]. There were no clinically important differences regarding quality of life, MD -0.01; 95% CI: -0.06 to 0.05, Fig 4, but because of the limited number of participants the certainty of evidence was rated as low quality. Table 2.

**Fig 5:**
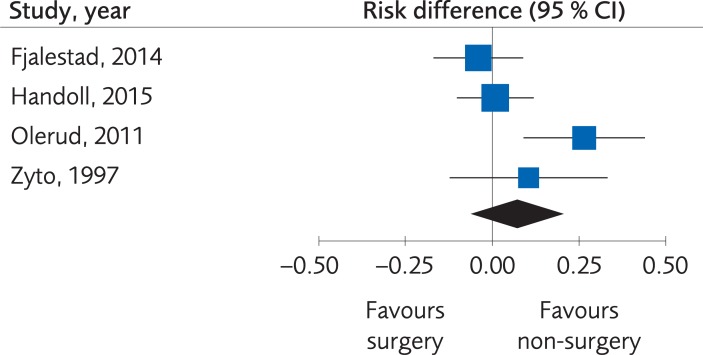
Meta-analysis of randomized controlled trials comparing different methods for internal fixation with treatment with a sling: results of major complications, in elderly patients with proximal humerus fractures at one-year follow-up.

Minor complications were reported by two RCTs and one Non-R study, but the number of events was too small to determine differences (15 events for operative treatment and 4 events for the non-operative treatment). Major complications were reported in in four RCTs and one Non-R study, but the certainty of evidence was rated as low quality, since there was a risk of bias and inconsistency. No clinically important differences were found, RD 0.07; 95% CI: -0.06 to 0.20, Fig 5.

### Comparison of different options for surgical treatment–Proximal humerus fractures

#### Study characteristics

Twenty-five trials, nine RCTs (572 participants) and sixteen Non-R studies (1 663 participants) were included that compared different surgical treatment options in elderly patients with PHF. The subjects were predominantly female. Out of these studies, fifteen were conducted in Europe, seven in Asia, one in USA, one in South America and one in Oceania. The treatment methods analyzed were surgical treatments with different kinds of internal fixation with plates, different kinds of plates with or without medial support, different kinds of internal fixation compared to prosthesis, plate fixation compared to intramedullary nail, RSA compared to HA, different kinds of fixation of the tubercles during arthroplasty and different kinds of intramedullary nails.

#### Comparison of plates, nails and prostheses

*Results*. When different kinds of plate fixation with or without angle-stable screws, plate fixation with or without screws supporting the fracture medially, internal fixation and arthroplasty were compared, there were not enough data to present results regarding differences in clinical result, quality of life or complications. The certainty of evidence was rated as very low (Table 3).

**Fig 6:**
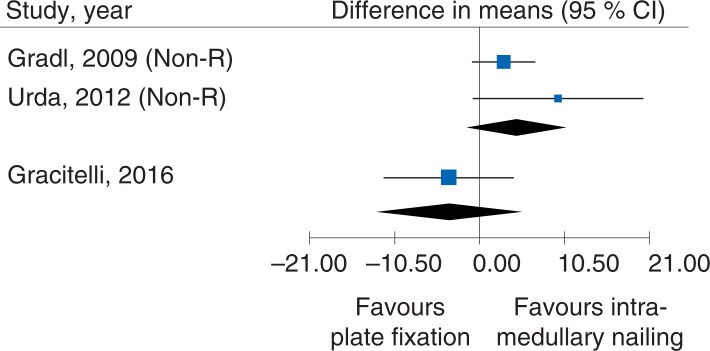
Meta-analysis of a randomized controlled trial and two Non-R studies comparing different methods for plate fixation with intramedullary nailing: results of functional outcomes, in elderly patients with proximal humerus fractures at one-year follow-up. The study of Konrad et al., 2012 could not be illustrated in the meta-analysis, since only boxplot was presented.

One RCT (65 participants) (using DASH as outcome) and two Non-R studies (193 participants) (using Constant score as outcome) compared plate fixation with intramedullary nailing. No clinically important differences were seen, MD_RCT_, -3.8 (95% CI: -11.85 to 4.25) and MD_cohorts_: 4.51 (95% CI: -0.99 to 10.1), Fig 6. The certainty of evidence was rated as low, due to the low number of trials and participants, and indirectness (lack of generalizability between the study designs). Analysis of quality of life and complications also failed due to too few observations.

#### Comparison of hemiarthroplasty vs. Reverse shoulder arthroplasty

**Fig 7:**
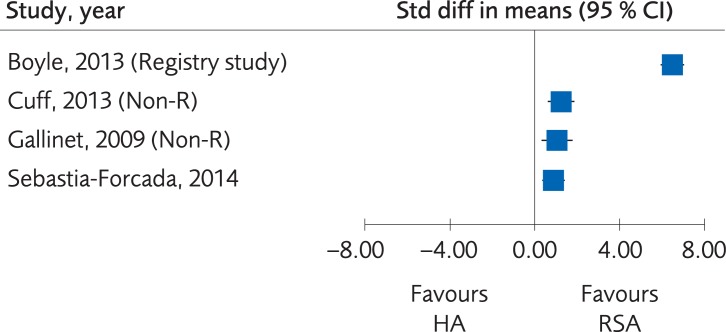
Meta-analysis of a randomized controlled trial and two Non-R studies and one registry study comparing hemiarthroplasty (HA) with reverse shoulder arthroplasty (RSA): Results of functional outcome, in elderly patients with proximal humerus fractures at one to five-year follow-up.

*Results*. One RCT, two Non-R studies and one registry study compared RSA with HA regarding functional outcome (OSS, DASH or American Shoulder and Elbow Surgeons Standardized Shoulder Assessment Form (ASES)). The combined results [61–63, 64] showed a statistically significant difference in favor of RSA, MD_RCT_ 6.9 (95% CI: 3.0 to 10.8), Fig 7, but this difference is not clinically significant. The certainty of evidence, however, was rated as very low due to the Non-R studies included and limitations in study design (Table 3).

### Comparison of different non-surgical treatments–Proximal humerus fractures

#### Study characteristics

One RCT (49 participants) was included that compared different non-surgical regimens. The subjects were predominantly female, and the study was conducted in Europe. The treatment methods analyzed were early and late immobilization after HA.

*Results*. The study did not provide enough data for determination of differences regarding functional outcome and complications. Quality of life was not evaluated, and certainty of evidence was rated as very low (Table 4).

### Comparison of different options for surgical treatment–Diaphyseal humerus fractures

#### Study characteristics

Only one Non-R study (511 participants) was identified that compared different surgical treatments options in elderly with midshaft humerus fractures. The subjects were predominantly female, and the study was conducted in the USA.

*Results*. The study compared plate fixation and nailing regarding complications for elderly with humeral shaft fractures [79]. Due to only one study with a low number of participants, the certainty of evidence was rated as very low quality. No studies corresponding to our inclusion criteria evaluated plate fixation versus nailing for elderly with humeral shaft fractures regarding clinical function or quality of life. The certainty of evidence was rated as very low.

### Comparison of different options for surgical treatment–Distal humerus fractures (supracondylar fractures)

#### Study characteristics

One controlled randomized study (40 participants) and one Non-R study (32 participants) compared different surgical treatments options in elderly with distal humerus fractures. The subjects were predominantly female, and the studies were conducted in Europe and Canada.

*Results*. One RCT compared plate fixation with total elbow arthroplasty regarding clinical function and complications, for displaced intra-articular distal humeral fractures in elderly patients [80]. The certainty of evidence was rated as very low. No studies corresponding to the project's criteria evaluated ORIF versus total elbow arthroplasty for elderly with distal humerus fractures regarding quality of life. The certainty of evidence was rated as very low.

One Non-R study compared early versus delayed total elbow replacement for distal humeral fractures in the elderly, regarding clinical function and complications [81]. The certainty of evidence was rated as very low quality. No studies corresponding to our project's inclusion criteria evaluated early versus delayed treatment of total elbow arthroplasty for elderly with distal humerus fractures regarding quality of life. The certainty of evidence was rated as very low.

### Health economic studies

Three economic evaluations were identified that evaluated treatments of PHF [47–49]. All three studies were assessed to have moderate quality, but the study by Nwachukwu and colleagues [48] showed low transferability to Swedish conditions. No studies investigated diaphyseal or distal fractures of the humerus.

Two of the cost-utility analyses compared surgical to non-surgical treatment for humerus fractures. The PROHFER study, by Corbacho et al., 2016 [47] showed that surgical treatment was more expensive and yielded worse outcome as measured by the EQ-5D (although not statistically significant result) when compared to non-surgical treatment. At a willingness to pay per QALY of GBP 30,000, their estimations showed a probability of only 23% that surgical treatment would be a cost-effective alternative compared to the non-surgical treatment. In contrast, Fjalestad et al., 2010 [49] reported initially higher costs for surgical treatment compared to non-surgical treatment but these differences evened out when a longer-term analysis was performed due to a larger number of out-patient visits for the non-operatively treated group after hospital dismissal. In addition, Fjalestad et al., 2010 [49] showed a small but not statistically significant difference in QALYs in favor of surgery (0.009 QALYs, 95% CI -0.025 to 0.042). Thus, the authors concluded that surgery was both less costly and more effective from a health care perspective. Nevertheless, the costs for surgery increased when including production losses in the analysis. In this scenario, the cost per QALY increased to approximately EURO 231,000 which corresponds to USD 340,000.

### Cost analysis

Intervention costs for treatment of PHF are shown in Table 5. The intervention costs ranged from USD 40 for treatment with immobilization in a sling, to USD 7 728 for surgical treatment with RSA, which was the most expensive treatment method. The treatment cost for IM nail fixation was slightly lower than for plate fixation due to a shorter operation time. The higher costs associated with HA and RSA are explained by higher implant costs, longer operation time and longer inpatient care. Surgery with HA and RSA was estimated to 120 minutes [52, 64], while the operation time for plate fixation and IM nail fixation was estimated to 100 and 60 minutes respectively [43, 51, 66, 73, 74, 82–85]. The time in inpatient care was estimated to be two days for plate fixation and IM nail fixation, and three days for HA and RSA.

**Table 5:** Intervention costs per treatment of proximal humerus fractures in a Swedish setting. Estimation performed within the context of a HTA analysis performed by the Swedish Agency for Health Technology Assessment and Assessment of Social Services (SBU).

	Intervention cost (USD 2016)				
Resources	Sling	Plate fixation	Intramedullary (IM) nail fixation	Hemiarthroplasty (HA)	Reverse shoulder arthroplasty (RSA)
Material (consumables and implants)	2	489	489	1363	3518
Time in the operating theatre including fixed equipment + overhead costs		890	762	953	953
Orthopaedic surgeon	24	286	191	333	333
Assisting orthopaedic surgeon		238	143	286	286
Anaesthesist		155	155	155	155
Anaesthetic nurse		318	272	340	340
Surgical nurse		318	272	340	340
Operation assistant		222	191	238	238
Out-patient clinic nurse	14	0	0	0	0
Inpatient care due to operation	0	1043	1043	1565	1565
Total	40	3959	3518	5573	7728

### Registry analysis

**Fig 8:**
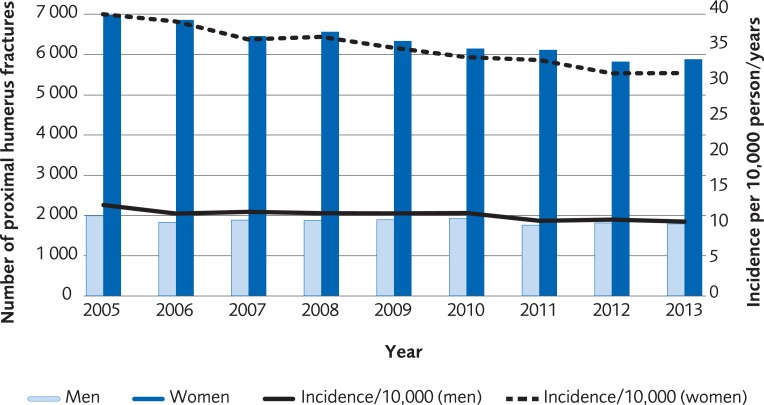
Incidence per 10,000 person-years of proximal humerus fractures in the Swedish population ≥50 years old, between 2005 and 2013 according to registry data from the Swedish Board of National Health and Welfare.

**Fig 9:**
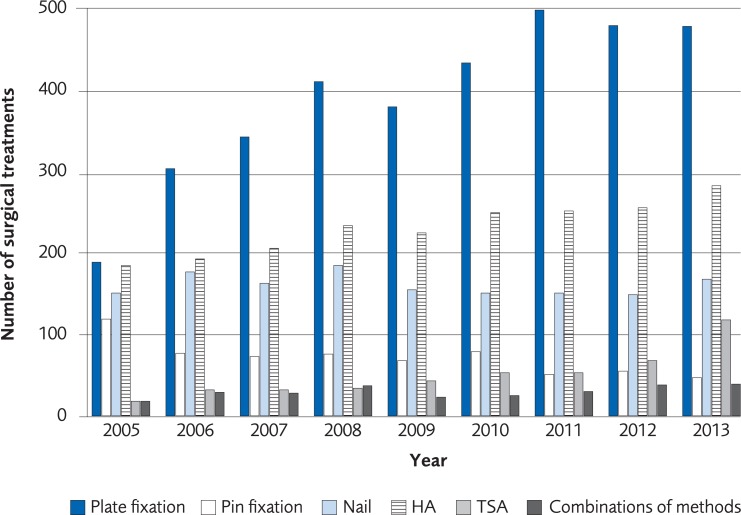
The most common surgical treatments of proximal humerus fracture in women, ≥50 years old, between 2005 to 2013 in Sweden according to registry data from the Swedish Board of National Health and Welfare.

**Fig 10:**
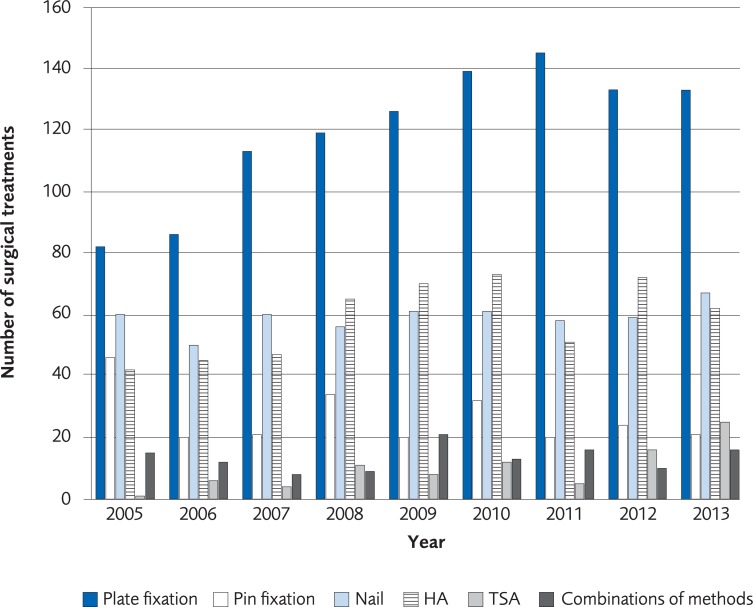
The most common surgical treatments of proximal humerus fracture in men, ≥50 years old, between 2005 to 2013 in Sweden according to registry data from the Swedish Board of National Health and Welfare.

The analysis was performed on registry data from 57,075 women and 16,774 men over the age of 50 recorded in the Swedish National Patient Registry with a PHF during the period 2005 to 2013. The incidence of a PHF in patients over 50 years old was 33/10,000-person years among women in 2005 and 31/10,000-person years in 2013. The incidence of a PHF in men in the same age group was 12/10,000-person years in 2005 and 10/10,000 in 2013 (Fig 8). The proportion of patients undergoing surgical treatment increased by 5.5% for women and 2.5% for men during 2005–2013. The most common method for surgical treatment in 2005 was plate fixation and its use increased until 2011 (Fig 9, for women, Fig 10 for men). The number of total shoulder arthroplasty (TSA) increased between 2011 and 2013 from 58 to 143, which represents an increase of 147%. Risk of death within 30 days from the PHF was 2.1%.

## Discussion

The present HTA analysis presents the current literature on treatment of humeral fractures including RCTs and cohort studies performed on patient over 60 years of age. The main finding is that there was no proven benefit with surgical intervention of moderately displaced PHF compared to non-operative treatment with immobilization in a sling. Moreover, the health economic evaluation showed that surgical treatment of a proximal humeral fracture was considerably more expensive (USD 3478 to USD 7688) than non-operative treatment. Many areas under investigation in this HTA analysis lacks sufficient scientific data to express any certitude regarding efficacy, effectiveness, complications and cost-effectiveness.

Our findings regarding functional outcome after PHF are supported by a review published by Launonen et al., 2015 [4]. They found no benefit of surgical treatment in patients over 60 years of age, with 3- or 4-parts PHF as compared to non-surgical treatment. Launonen and colleagues found more complications in the group of surgically treated patients than in the non-operative group. Equal findings regarding functional outcome and complications were reported in a systematic review by Beks et al., 2018 [86]. According to definitions used in our literature review, we consider the available evidence too scarce to draw any qualified conclusions regarding comparisons of complications after operative or non-operative treatment of PHF. In contrast to Launonen and Beks, we also included studies regarding diaphyseal and distal humerus fractures in our literature search. Other authors support our opinion that there is a lack of scientific data regarding treatment of diaphyseal and distal humeral fractures, especially in elderly patients [87–89].

In accordance with our finding, Sabharwal et al., 2016 [90] and Beks et al., 2018 [86] concluded in their reviews that there was no overall benefit of surgical treatment of proximal humeral fractures. These reviews used Constant score as the most important outcome measure, as opposed to our study where we used DASH as the preferred primary outcome. A sub group analysis of different fracture patterns was performed in the study by Sabharwal., 2016 [90], indicating higher quality of life for patients with 4-part fractures, i.e. more complex fractures, when treated operatively. Interestingly, fractures of high complexity have been excluded from clinical trials, e.g. Olerud et al., 2011 [51, 52] and Handoll et al., 2015 [50], with the explanation that the indication for surgery was absolute. Moreover, the definitions of inclusion criteria regarding fracture types and degrees of displacement vary widely between studies. In some studies, radiographic inclusion criteria were not clearly described. One should refrain from generalizing findings from studies investigating moderately displaced fractures to populations suffering from complex fracture patterns. We support Sabharwal et al., 2016 [90] in their opinion that future studies must be very strict and clear when defining and describing inclusion criteria regarding fracture classification and degree of displacement.

In the present systematic review, the majority of data was collected from follow-up at one year. There is a paucity of adequate publications of short- mid and long-term follow-ups and more studies must be performed to discern potential differences on different time horizons. According to Handoll et al., 2017 [91], who reported no differences in reoperation rates between the 2-year and 5-year follow-up of their multicenter study comparing surgery and non-surgical treatment, there is reason to believe that no more than 2 years is necessary for final follow-up. In their study they presented slight improvement as measured by the OSS, equally large in both groups, between the 2- and 5 years follow-up, although the improvement was statistically but not clinically significant [91].

There is a lack of studies regarding the cost-effectiveness of treatment of humeral fractures. The few studies that have been published [47–49] have had short time perspectives and showed conflicting results. This highlights the need for more well-designed cost-effectiveness studies. The costs to be included in such an analysis may differ from country to country and between patient populations. In the age group studied in this report, prolonged hospital stays, temporary stays in home for the elderly, and home care should be included in a cost-effectiveness analysis, whereas in a younger population sick-leave and rehabilitation costs may be of larger importance. Long term analysis is important in future studies, since an initially more expensive treatment method may be less expensive in the long run if out-patient care visits and complications can be avoided [49]. It appears conflicting, that the incidence of expensive fracture surgery for PHF is increasing despite the lack of scientific evidence of its effectiveness. Surgical treatment that is not cost-effective should not be performed. A retrospective analysis of current surgical practice in a British setting, confirmed our findings that surgical treatment was more expensive than non-operative treatment [92]. They stated that extensive resources could be saved if non-operative treatment was chosen instead. However, future studies on both clinical effectiveness and cost effectiveness are needed to clarify the relationship between costs and effects of treatment methods used for humerus fractures.

The proportion of operative treatments for PHF in the elderly has showed an increasing trend in Sweden over the last years, as demonstrated by our data retrieved from the Swedish National Patient Registry. Similar trends have been observed in the USA. Bell et al., 2011 [7] presented data from 1999 to 2005 with rising surgery rates of PHF [7]. Rosas et al., 2016 [93] also reported similar findings and their study supports our findings of a rising incidence of total shoulder arthroplasty following PHF. Another confirmation of our findings of patterns of surgery trends can be found in the publication from Khatib et al., 2014 [94] who presented data on the same age group as we did.

A registry-based study from Sumrein et al., 2017 [8] reported a steeply rising incidence of PHF in Sweden between 2001–2012. This is contrary to our findings of a slightly decreasing incidence of PHF 2005–2013. Sumrein et al., 2017 used registry data from the Swedish National Patient registry, as in our systematic review, and included all patients over 18 years of age. Data was used from the out- and inpatient registry, and data was presented for separate age groups individually. The difference in results compared to our study may be explained that the out-patient registry started in 2001 and data from the first few years of registrations may not have been complete. Missing data during 2001–2005 may have been misinterpreted as increasing incidence. A different age stratification between our and Sumrein’s studies may also be an explanation for differences in findings. The Swedish Patient registry has a good validity regarding in-patient care [95] but to the best of our knowledge, the out-patient registry has not been validated regarding ICD10 codes for humerus fractures. Validation studies of any registry are important to confirm accuracy of findings.

This systematic review shows that there is very low certainty of evidence for treatment options for diaphyseal or distal fractures of the humerus in elderly patients. The absence of conclusive evidence should not be interpreted as evidence of lack of effectiveness. Rather, it means that further research is very likely to have an important impact.

## Limitations

Some limitations of our systematic review should be noted. Despite the large number of publications on the treatment of humeral fractures, few of them meet the scientific standards to be included in our review. The low number of included trials–in combination with the heterogeneous interventions, comparators, and populations–made statistical tests of publication bias unreliable. We also solely had to rely on the information available in the included reports. Some reports did not, for instance, clearly indicate the severity of the fracture, the quality of the bone, when the surgery was performed in relation to the day of the fracture, or the number of individuals included in the analyses at one-year follow-up. In such situations, we assumed that all randomized participants were included, which might not always have been the case. Whether different modalities of non-operative treatment, including physiotherapy, could have influenced the outcome cannot be determined. We only reported outcomes at one-year follow-up, due to low number of included trials that reported on shorter times.

Evaluation of radiologic outcome was not performed in this systematic review and we have not included radiologic malunion in the presentation of complications in our report. On the other hand, we believe that a clinically significant malunion is likely to have been expressed as poor outcome. Other authors have also refrained from analyzing radiographic data of humerus fractures [4, 50, 90].

The registry data used for the analysis of surgical practice in Sweden has limitations since the fracture code (ICD10) does not allow specification of fracture severity and the procedural code e.g. does not discriminate between different types of arthroplasties. Detailed validation studies of the Swedish National Patient Registry have not been performed for fracture diagnoses and treatments.

An obvious strength with our study is its size and its coverage, it is methodologically sound and robust, and the production of all results have continuously been reviewed by expertise from the Swedish Agency for Health Technology Assessment and Assessment of Social Services, SBU. Another strength is that diaphyseal and distal humeral fractures also have been included in this systematic review.

## Conclusions

Despite the limitations and the relatively few studies included, we conclude that surgical treatment of moderately displaced fractures of the proximal humerus in the elderly yields no clear benefit compared to the less costly non-surgical treatment option.

Based on these data one should have a restrictive attitude to surgical treatment of PHF. However, each patient should be treated individually rather than on a statistical basis and consideration has to be taken to fracture pattern and specific needs of the patient. For fractures of the distal parts of the humerus, too few studies have been published to draw meaningful conclusions in this context.

In future research, one should focus on methodologically well-conducted prospective comparative studies to evaluate the rate of functional recovery and outcomes associated with treatment methods, for humerus fractures, in which the population is clearly described regarding age, sex, bone quality and severity of the fracture. To contribute to higher quality of evidence, the studies need to have sufficiently number of individuals included, long follow-up (more than a year) and use validated measurement methods. Future studies should also report aspects of quality of life with validated instruments and include cost-effectiveness analyses.

## Supporting information

S1 Appendix Search strategy. (PDF)

S2 Appendix Included publication. (PDF)

S3 Appendix Excluded publications. (PDF)

S4 Appendix PRISMA check-list. (PDF)

S5 Appendix Cost analysis. (PDF)

S6 Appendix Description of studies. (PDF)
