# Supplementary material for: Treatment of humerus fractures in the elderly: A systematic review covering effectiveness, safety, economic aspects and evolution of practice
Source: PLoS One. 2018 Dec 13;13(12):e0207815. doi: 10.1371/journal.pone.0207815 (PMC6292626; doi:10.1371/journal.pone.0207815)
Supplement: S2 Appendix — (PDF) [file pone.0207815.s002.pdf]

S2 Appendix.

Publications included in a HTA analysis of humerus fractures, performed by the Swedish Agency for Health Technology Assessment and Assessment of Social Services

Table of Contents

Included publications – Effectiveness and complications of treatments 1

Randomized controlled trials (18 trials)... 1

Non-randomized control trials (21 trials) 3

Included publications – health economic evaluations.. 6

Included publications – Effectiveness and complications of treatments

Randomized controlled trials (18 trials)

1. Agorastides, I., C. Sinopidis, M. El Meligy, Q. Yin, P. Brownson and S. P. Frostick (2007). "Early versus late mobilization after hemiarthroplasty for proximal humeral fractures." J Shoulder Elbow Surg 16(3 Suppl): S33-38.

2. Boons, H. W., J. H. Goosen, S. van Grinsven, J. L. van Susante and C. J. van Loon (2012). "Hemiarthroplasty for humeral four-part fractures for patients 65 years and older: a randomized controlled trial." Clin Orthop Relat Res 470(12): 3483-3491.

3. Buecking, B., J. Mohr, B. Bockmann, R. Zettl and S. Ruchholtz (2014). "Deltoid-split or deltopectoral approaches for the treatment of displaced proximal humeral fractures?" Clin Orthop Relat Res 472(5): 1576-1585.

4. Chen, H., X. Ji, Y. Gao, L. Zhang, Q. Zhang, X. Liang and P. Tang (2016). "Comparison of intramedullary fibular allograft with locking compression plate versus shoulder hemi-arthroplasty for repair of osteoporotic four-part proximal humerus fracture: Consecutive, prospective, controlled, and comparative study." Orthop Traumatol Surg Res 102(3): 287-292.

5. Fialka, C., P. Stampfl, S. Arbes, P. Reuter, G. Oberleitner and V. Vecsei (2008). "Primary hemiarthroplasty in four-part fractures of the proximal humerus: randomized trial of two different implant systems." J Shoulder Elbow Surg 17(2): 210-215.

6. Fjalestad, T. and M. O. Hole (2014). "Displaced proximal humeral fractures: operative versus non-operative treatment--a 2-year extension of a randomized controlled trial." Eur J Orthop Surg Traumatol 24(7): 1067-1073.

7. Fjalestad, T., M. O. Hole, I. A. Hovden, J. Blucher and K. Stromsoe (2012). "Surgical treatment with an angular stable plate for complex displaced proximal humeral fractures in elderly patients: a randomized controlled trial." J Orthop Trauma 26(2): 98-106.

8. Gracitelli, M. E., E. A. Malavolta, J. H. Assuncao, K. E. Kojima, P. R. Dos Reis, J. S. Silva, A. A. Ferreira Neto and A. J. Hernandez (2016). "Locking intramedullary nails compared with locking plates for two- and three-part proximal humeral surgical neck fractures: a randomized controlled trial." J Shoulder Elbow Surg 25(5): 695-703.

9. Handoll, H., S. Brealey, A. Rangan, A. Keding, B. Corbacho, L. Jefferson, L. H. Chuang, L. Goodchild, C. Hewitt and D. Torgerson (2015). "The ProFHer (PROximal fracture of the humerus: Evaluation by randomisation) trial – A pragmatic multicentre randomized controlled trial evaluating the clinical effectiveness and cost-effectiveness of surgical compared with non-surgical treatment for proximal fracture of the humerus in adults." Health Technology Assessment 19(24): 1-279.

10. Liu, Z. Z., G. M. Zhang and T. Ge (2011). "Use of a proximal humeral internal locking system enhanced by injectable graft for minimally invasive treatment of osteoporotic proximal humeral fractures in elderly patients." Orthop Surg 3(4): 253-258.

11. Lopiz, Y., J. Garcia-Coiradas, C. Garcia-Fernandez and F. Marco (2014). "Proximal humerus nailing: a randomized clinical trial between curvilinear and straight nails." J Shoulder Elbow Surg 23(3): 369-376.

12. McKee, M. D., C. J. Veillette, J. A. Hall, E. H. Schemitsch, L. M. Wild, R. McCormack, B. Perey, T. Goetz, M. Zomar, K. Moon, S.

Mandel, S. Petit, P. Guy and I. Leung (2009). "A multicenter, prospective, randomized, controlled trial of open reduction--internal fixation versus total elbow arthroplasty for displaced intra-articular distal humeral fractures in elderly patients." J Shoulder Elbow Surg 18(1): 3-12.

13. Olerud, P., L. Ahrengart, S. Ponzer, J. Saving and J. Tidermark (2011). "Hemiarthroplasty versus nonoperative treatment of displaced 4-part proximal humeral fractures in elderly patients: a randomized controlled trial." J Shoulder Elbow Surg 20(7): 1025-1033.

14. Olerud, P., L. Ahrengart, S. Ponzer, J. Saving and J. Tidermark (2011). "Internal fixation versus nonoperative treatment of displaced 3-part proximal humeral fractures in elderly patients: a randomized controlled trial." J Shoulder Elbow Surg 20(5): 747-755.

15. Sebastia-Forcada, E., R. Cebrian-Gomez, A. Lizaur-Utrilla and V. Gil-Guillen (2014). "Reverse shoulder arthroplasty versus hemiarthroplasty for acute proximal humeral fractures. A blinded, randomized, controlled, prospective study." J Shoulder Elbow Surg.

16. Voigt, C., A. Geisler, P. Hepp, A. P. Schulz and H. Lill (2011). "Are polyaxially locked screws advantageous in the plate osteosynthesis of proximal humeral fractures in the elderly? A prospective randomized clinical observational study." J Orthop Trauma 25(10): 596-602.

17. Zhang, L., J. Zheng, W. Wang, G. Lin, Y. Huang, J. Zheng, G. A. Edem Prince and G. Yang (2011). "The clinical benefit of medial support screws in locking plating of proximal humerus fractures: a prospective randomized study." Int Orthop 35(11): 1655-1661.

18. Zyto, K., L. Ahrengart, A. Sperber and H. Tornkvist (1997). "Treatment of displaced proximal humeral fractures in elderly patients." J Bone Joint Surg Br 79(3): 412-417.

Non-randomized control trials (21 trials)

1. Boyle, M. J., S. M. Youn, C. M. Frampton and C. M. Ball (2013). "Functional outcomes of reverse shoulder arthroplasty compared

with hemiarthroplasty for acute proximal humeral fractures." J Shoulder Elbow Surg 22(1): 32-37.

2. Chen, F., Z. Wang and T. Bhattacharyya (2013). "Outcomes of nails versus plates for humeral shaft fractures: a Medicare cohort study." J Orthop Trauma 27(2): 68-72.

3. Cuff, D. J. and D. R. Pupello (2013). "Comparison of hemiarthroplasty and reverse shoulder arthroplasty for the treatment of proximal humeral fractures in elderly patients." J Bone Joint Surg Am 95(22): 2050-2055.

4. Dietz, S. O., P. Broos, S. Nijs, A. (2012). "Suture fixation versus cable cerclage of the tuberosities in shoulder arthroplasty-clinical and radiologic results." Archives of Orthopaedic and Trauma Surgery 132(6): 793-800.

5. Gallinet, D., P. Clappaz, P. Garbuio, Y. Tropet and L. Obert (2009). "Three or four parts complex proximal humerus fractures: hemiarthroplasty versus reverse prosthesis: a comparative study of 40 cases." Orthop Traumatol Surg Res 95(1): 48-55.

6. Gradl, G., A. Dietze, M. Kaab, W. Hopfenmuller and T. Mittlmeier (2009). "Is locking nailing of humeral head fractures superior to locking plate fixation?" Clin Orthop Relat Res 467(11): 2986-2993.

7. Hauschild, O., G. Konrad, L. Audige, P. de Boer, S. M. Lambert, R. Hertel and N. P. Sudkamp (2013). "Operative versus non-operative treatment for two-part surgical neck fractures of the proximal humerus." Arch Orthop Trauma Surg 133(10): 1385-1393.

8. Innocenti, M., C. Carulli, R. Civinini, F. Matassi, M. Tani, F. Muncibi, A. (2013). "Displaced fragility fractures of proximal humerus in elderly patients affected by severe comorbidities: Percutaneous fixation and conservative treatment." Aging Clinical and Experimental Research 25(4): 447-452.

9. Konrad, G., L. Audige, S. Lambert, R. Hertel and N. P. Sudkamp (2012). "Similar outcomes for nail versus plate fixation of three-part proximal humeral fractures." Clin Orthop Relat Res 470(2): 602-609.

10. Konrad, G., A. Hirschmuller, L. Audige, S. Lambert, R. Hertel and N. P. Sudkamp (2012). "Comparison of two different locking plates for two-, three- and four-part proximal humeral fractures--results of an international multicentre study." Int Orthop 36(5): 1051-1058.

11. Loew, M., S. Heitkemper, D. Parsch, S. Schneider and M. Rickert (2006). "Influence of the design of the prosthesis on the outcome after hemiarthroplasty of the shoulder in displaced fractures of the head of the humerus." J Bone Joint Surg Br 88(3): 345-350.

12. Ortmaier, R., V. Filzmaier, W. Hitzl, R. Bogner, T. Neubauer, H. Resch and A. Auffarth (2015). "Comparison between minimally invasive, percutaneous osteosynthesis and locking plate osteosynthesis in 3-and 4-part proximal humerus fractures." BMC Musculoskelet Disord 16: 297.

13. Ortmaier, R., G. Mattiassich, M. Pumberger, W. Hitzl, P. Moroder, A. Auffarth and H. Resch (2015). "Comparison between reverse shoulder arthroplasty and Humerusblock in three- and four-part proximal humerus fractures in elderly patients." Int Orthop 39(2): 335-342.

14. Peng, C. H., W. T. Wu, T. C. Yu, L. C. Chen, S. H. Hsu, S. T. Kwong, T. K. Yao, K. C. Wu, P. C. Shao, J. H. Wang and I. H. Chen (2012). "Surgical treatment for proximal humeral fracture in elderly patients with emphasis on the use of intramedullary strut allografts." Tzu Chi Medical Journal 24(3): 131-135.

15. Prasad, N. and C. Dent (2008). "Outcome of total elbow replacement for distal humeral fractures in the elderly: a comparison of primary surgery and surgery after failed internal fixation or conservative treatment." J Bone Joint Surg Br 90(3): 343-348.

16. Schai, P., A. Imhoff and S. Preiss (1995). "Comminuted humeral head fractures: a multicenter analysis." J Shoulder Elbow Surg 4(5): 319-330.

17. Shi, H. F., J. Xiong, Y. X. Chen, J. F. Wang, S. F. Wang, Z. J. Chen and Y. Qiu (2011). "Management of proximal humeral fractures in elderly patients with uni- or polyaxial locking osteosynthesis system." Arch Orthop Trauma Surg 131(4): 541-547.

18. Spross, C., A. Platz, M. Erschbamer, T. Lattmann and M. Dietrich (2012). "Surgical treatment of Neer Group VI proximal humeral fractures: retrospective comparison of PHILOS(R) and hemiarthroplasty." Clin Orthop Relat Res 470(7): 2035-2042.

19. Urda, A., A. Gonzalez, A. Colino, Y. Lopiz, C. Garcia-Fernandez and F. Marco (2012). "Management of displaced surgical neck fractures of the humerus: health related quality of life, functional and radiographic results." Injury 43 Suppl 2: S12-19.

20. Wu, X., S. H. Li, Z. D. Cai and L. M. Lou (2013). "Modified hemiarthroplasty for four-part fractures of the proximal humerus." ANZ J Surg 83(3): 165-170.

21. Yan, D., Y. Soon, A. Author, N. P. (2012). "Comparative study of T-plates and locking plates in the management of displaced proximal humeral fractures." Current Orthopaedic Practice 23(4): 351-355.

Included publications – health economic evaluations

1. Corbacho, B., A. Duarte, A. Keding, H. Handoll, L. H. Chuang, D. Torgerson, S. Brealey, L. Jefferson, C. Hewitt, A. Rangan, A. (2016) "Cost effectiveness of surgical versus nonsurgical treatment of adults with displaced fractures of the proximal humerus: Economic evaluation alongside the profher trial." Bone and Joint Journal 98B (2): 152-159.

2. Fjalestad, T., M. O. Hole, J. J. Jorgensen, K. Stromsoe and I. S. Kristiansen (2010). "Health and cost consequences of surgical versus conservative treatment for a comminuted proximal humeral fracture in elderly patients." Injury 41(6): 599-605.

3. Nwachukwu BU, Schairer WW, McCormick F, Dines DM, Craig EV, Gulotta LV, (2016). "Arthroplasty for the surgical management of complex proximal humerus fractures in the elderly: A cost-utility analysis. Journal of Shoulder and Elbow Surgery.";25(5):704-13.
