# Supplementary material for: Treatment of humerus fractures in the elderly: A systematic review covering effectiveness, safety, economic aspects and evolution of practice
Source: PLoS One. 2018 Dec 13;13(12):e0207815. doi: 10.1371/journal.pone.0207815 (PMC6292626; doi:10.1371/journal.pone.0207815)
Supplement: S3 Appendix — (PDF) [file pone.0207815.s003.pdf]

S3 Appendix.

Publications excluded for the purpose of the HTA analysis of humerus fractures performed by the Swedish Agency for Health Technology Assessment and Assessment of Social Services

Table of Contents

Excluded publications – Effectiveness and complications of treatments............2
Reasons: Not relevant studies 2
Randomized controll trials (80 trials).. 2
Non-randomized control trials (44 trials) 12
Reasons: Studies with high risk of bias 17
Randomized control trials (13 trials) 17
Non-randomized control trials (30 trials) 19
Studies excluded, reason: not involving humerus fractures...........................22
Randomized control trials (31 trials) 22
Non-randomized control trials (10 trials) 26
Excluded publications – Health economic evaluations......................................27
Reasons: Studies not involving humerus fractures..27
Reasons: Studies that only studied resource use or costs, i.e. were not full health economic evaluations...28

Excluded publications – Effectiveness and complications of treatments

Reasons: Not relevant studies

Randomized controll trials (80 trials)

1. Ahmed, A. R., T. Sweed and A. Wanas (2008) "The role of cancellous screw with tension band fixation in the treatment of displaced olecranon fractures, a comparative study." European journal of orthopaedic surgery & traumatology: orthopedie traumatologie 18, 571-576 DOI: 10.1007/s00590-008-0355-0.

2. Alkhayarin, M. and M. N. Said (2012) "Third tubular versus 3.5-mm dynamic compression plates for fixation of diaphyseal fractures of the ulna: A prospective randomized trial." European journal of orthopaedic surgery & traumatology: orthopedie traumatologie 22, 561-564 DOI: 10.1007/s00590-011-0882-y.

3. Benegas, E., A. A. Ferreira Neto, M. E. Gracitelli, E. A. Malavolta, J. H. Assuncao, S. Prada Fde, R. Bolliger Neto and R. Mattar, Jr. (2014). "Shoulder function after surgical treatment of displaced fractures of the humeral shaft: a randomized trial comparing antegrade intramedullary nailing with minimally invasive plate osteosynthesis." J Shoulder Elbow Surg 23(6): 767-774.

4. Bong, M. R., K. A. Egol, M. Leibman and K. J. Koval (2006). "A comparison of immediate postreduction splinting constructs for controlling initial displacement of fractures of the distal radius: a prospective randomized study of long-arm versus short-arm splinting." J Hand Surg Am 31(5): 766-770.

5. Casteleyn, P. P., F. Handelberg and P. Haentjens (1992). "Biodegradable rods versus Kirschner wire fixation of wrist fractures." Journal of Bone and Joint Surgery - Series B 74(6): 858-861.

6. Chan, Y. H., T. L. Foo, C. J. Yeo and W. Y. Chew (2014). "Comparison between cast immobilization versus volar locking plate fixation of distal radius fractures in active elderly patients, the Asian perspective." Hand Surg 19(1): 19-23.

7. Changulani, M., U. K. Jain and T. Keswani (2007). "Comparison of the use of the humerus intramedullary nail and dynamic compression plate for the management of diaphyseal fractures of the humerus. A randomised controlled study." Int Orthop 31(3): 391-395.

8. Chapman, J. R., M. B. Henley, J. Agel and P. J. Benca (2000). "Randomized prospective study of humeral shaft fracture fixation: intramedullary nails versus plates." J Orthop Trauma 14(3): 162-166.

9. Chappuis, J., P. Boute and P. Putz (2011). "Dorsally displaced extra-articular distal radius fractures fixation: Dorsal IM nailing versus volar plating. A randomized controlled trial." Orthop Traumatol Surg Res 97(5): 471-478.

10. Chen, X., P. Liu, X. Zhu, L. Cao, C. Zhang and J. Su (2013). "Design and application of nickel-titanium olecranon memory connector in treatment of olecranon fractures: a prospective randomized controlled trial." Int Orthop 37(6): 1099-1105.

11. Chen, X., S. C. Wang, L. H. Cao, G. Q. Yang, M. Li and J. C. Su (2011). "Comparison between radial head replacement and open reduction and internal fixation in clinical treatment of unstable, multi-fragmented radial head fractures." Int Orthop 35(7): 1071-1076.

12. Chiu, F. Y., C. M. Chen, C. F. J. Lin, W. H. Lo, Y. L. Huang and T. H. Chen (1997). "Closed humeral shaft fractures: A prospective evaluation of surgical treatment." Journal of Trauma - Injury, Infection and Critical Care 43(6): 947-951.

13. Cohen, M. S. and T. Frillman (1997). "Distal radius fractures: a prospective randomized comparison of fibreglass tape with QuickCast." Injury 28(4): 305-309.

14. Cooper, L., C. Mauffrey, P. Hull, M. Brewster, C. Lewis and P. Makrides (2008). "A short term functional outcome study comparing closed reduction percutaneous wire fixation with open reduction internal fixation for fractures of the distal radius: A pilot study." European Journal of Orthopaedic Surgery and Traumatology 18(8): 551-554.

15. D'Agostino, P. and O. Barbier (2013). "An investigation of the effect of AlloMatrix bone graft in distal radial fracture: a prospective randomised controlled clinical trial." Bone Joint J 95-b (11): 1514-1520.

16. Earnshaw, S. A., A. Aladin, S. Surendran and C. G. Moran (2002). "Closed reduction of colles fractures: comparison of manual manipulation and finger-trap traction: a prospective, randomized study." J Bone Joint Surg Am 84-a(3): 354-358.

17. Egol, K., M. Walsh, N. Tejwani, T. McLaurin, C. Wynn and N. Paksima (2008). "Bridging external fixation and supplementary Kirschner-wire fixation versus volar locked plating for unstable fractures of the distal radius: a randomised, prospective trial." J Bone Joint Surg Br 90(9): 1214-1221.

18. Egol, K. A., M. T. Sugi, C. C. Ong, N. Montero, R. Davidovitch and J. D. Zuckerman (2012). "Fracture site augmentation with calcium phosphate cement reduces screw penetration after open reduction-internal fixation of proximal humeral fractures." J Shoulder Elbow Surg 21(6): 741-748.

19. Ekrol, I., C. Hajducka, C. Court-Brown and M. M. McQueen (2008). "A comparison of RhBMP-7 (OP-1) and autogenous graft for metaphyseal defects after osteotomy of the distal radius." Injury 39 Suppl 2: S73-82.

20. Gebuhr, P., P. Holmich, T. Orsnes, M. Soelberg, M. Krasheninnikoff and A. G. Kjersgaard (1992). "Isolated ulnar shaft fractures. Comparison of treatment by a functional brace and long-arm cast." J Bone Joint Surg Br 74(5): 757-759.

21. Gil-Guillen, V. (2014) "Reverse shoulder arthroplasty versus hemiarthroplasty for acute proximal humeral fractures. A blinded, randomized, controlled, prospective study." Journal of shoulder and elbow surgery 23, 1419-1426 DOI: http://dx.doi.org/10.1016/j.jse.2014.06.035.

22. Grafstein, E., R. Stenstrom, J. Christenson, G. Innes, R. MacCormack, C. Jackson, K. Stothers and T. Goetz (2010). "A prospective randomized controlled trial comparing circumferential casting and splinting in displaced Colles fractures." Cjem 12(3): 192-200.

23. Grewal, R., B. Perey, M. Wilmink and K. Stothers (2005). "A randomized prospective study on the treatment of intra-articular distal radius fractures: open reduction and internal fixation with dorsal plating versus mini open reduction, percutaneous fixation, and external fixation." J Hand Surg Am 30(4): 764-772.

24. Gupta, A., D. Barei, A. Khwaja and D. Beingessner (2014). "Single-staged treatment using a standardized protocol results in functional motion in the majority of patients with a terrible triad elbow injury." Clin Orthop Relat Res 472(7): 2075-2083.

25. Gupta, R., A. Raheja and U. Modi (1999). "Colles' fracture: management by percutaneous crossed-pin fixation versus plaster of Paris cast immobilization." Orthopedics 22(7): 680-682.

26. Hageman, M. G., P. Jayakumar, J. D. King, T. G. Guitton, J. N. Doornberg and D. Ring (2014). "The factors influencing the decision making of operative treatment for proximal humeral fractures." J Shoulder Elbow Surg.

27. Hahnloser, D., A. Platz, M. Amgwerd and O. Trentz (1999). "Internal fixation of distal radius fractures with dorsal dislocation: pi-plate or two 1/4 tube plates? A prospective randomized study." J Trauma 47(4): 760-765.

28. Handoll, H. H., L. Goodchild, S. D. Brealey, N. C. Hanchard, L. Jefferson, A. Keding and A. Rangan (2014). "Developing, delivering and documenting rehabilitation in a multi-centre randomised controlled surgical trial: experiences from the ProFHER trial." Bone Joint Res 3(12): 335-340.

29. Hargreaves, D. G., S. J. Drew and R. Eckersley (2004). "Kirschner wire pin tract infection rates: a randomized controlled trial between percutaneous and buried wires." J Hand Surg Br 29(4): 374-376.

30. Harley, B. J., A. Scharfenberger, L. A. Beaupre, N. Jomha and D. W. Weber (2004). "Augmented external fixation versus percutaneous pinning and casting for unstable fractures of the distal radius--a prospective randomized trial." J Hand Surg Am 29(5): 815-824.

31. Helling, H. J., A. Prokop, H. U. Schmid, M. Nagel, J. Lilienthal and K. E. Rehm (2006). "Biodegradable implants versus standard metal fixation for displaced radial head fractures. A prospective, randomized, multicenter study." J Shoulder Elbow Surg 15(4): 479-485.

32. Hutchinson, D. T., K. N. Bachus and T. Higgenbotham (2000) "External fixation of the distal raclius: To predrill or not to predrill." Journal of hand surgery 25, 1064-1068 DOI: 10.1053/jhsu.2000.17866.

33. Hutchinson, D. T., G. O. Strenz and R. A. Cautilli (1995). "Pins and plaster vs external fixation in the treatment of unstable distal radial fractures. A randomized prospective study." J Hand Surg Br 20(3): 365-372.

34. Jongs, R. A., L. A. Harvey, T. Gwinn and B. R. Lucas (2012). "Dynamic splints do not reduce contracture following distal radial fracture: a randomised controlled trial." J Physiother 58(3): 173-180.

35. Juutilainen, T., H. Patiala, P. Rokkanen and P. Tormala (1995). "Biodegradable wire fixation in olecranon and patella fractures combined with biodegradable screws or plugs and compared with metallic fixation." Archives of Orthopaedic and Trauma Surgery 114(6): 319-323.

36. Kapoor, H., A. Agarwal and B. K. Dhaon (2000). "Displaced intra-articular fractures of distal radius: a comparative evaluation of results following closed reduction, external fixation and open reduction with internal fixation." Injury 31(2): 75-79.

37. Karantana, A., N. D. Downing, D. P. Forward, M. Hatton, A. M. Taylor, B. E. Scammell, C. G. Moran and T. R. Davis (2013). "Surgical treatment of distal radial fractures with a volar locking plate versus conventional percutaneous methods: a randomized controlled trial." J Bone Joint Surg Am 95(19): 1737-1744.

38. Karantana, A., B. E. Scammell, T. R. Davis and D. K. Whynes (2015). "Cost-effectiveness of volar locking plate versus percutaneous fixation for distal radial fractures: Economic evaluation alongside a randomised clinical trial." Bone Joint J 97-b (9): 1264-1270.

39. Kim, J. W., C. W. Oh, Y. S. Byun, J. J. Kim and K. C. Park (2015). "A prospective randomized study of operative treatment for noncomminuted humeral shaft fractures: conventional open plating versus minimal invasive plate osteosynthesis." J Orthop Trauma 29(4): 189-194.

40. Kreder, H. J., J. Agel, M. D. McKee, E. H. Schemitsch, D. Stephen and D. P. Hanel (2006). "A randomized, controlled trial of distal radius fractures with metaphyseal displacement but without joint incongruity: closed reduction and casting versus closed reduction, spanning external fixation, and optional percutaneous K-wires." J Orthop Trauma 20(2): 115-121.

41. Krishnan, J., A. E. Wigg, R. W. Walker and J. Slavotinek (2003). "Intra-articular fractures of the distal radius: a prospective randomised controlled trial comparing static bridging and dynamic non-bridging external fixation." J Hand Surg Br 28(5): 417-421.

42. Krukhaug, Y., S. Ugland, S. A. Lie and L. M. Hove (2009). "External fixation of fractures of the distal radius: a randomized comparison of the Hoffman compact II non-bridging fixator and the Dynawrist fixator in 75 patients followed for 1 year." Acta Orthop 80(1): 104-108.

43. Kulshrestha, V., T. Roy and L. Audige (2011). "Dynamic vs static external fixation of distal radial fractures: A randomized study." Indian J Orthop 45(6): 527-534.

44. Lee, S. K., K. J. Kim, K. H. Park and W. S. Choy (2013). "A comparison between orthogonal and parallel plating methods for distal humerus fractures: a prospective randomized trial." Eur J Orthop Surg Traumatol.

45. Leung, F. and S. P. Chow (2003). "A prospective, randomized trial comparing the limited contact dynamic compression plate with the point contact fixator for forearm fractures." J Bone Joint Surg Am 85-a(12): 2343-2348.

46. Leung, F., Y. K. Tu, W. Y. Chew and S. P. Chow (2008). "Comparison of external and percutaneous pin fixation with plate fixation for intra-articular distal radial fractures. A randomized study." J Bone Joint Surg Am 90(1): 16-22.

47. Li, Y., C. Wang, M. Wang, L. Huang and Q. Huang (2011). "Postoperative malrotation of humeral shaft fracture after plating compared with intramedullary nailing." J Shoulder Elbow Surg 20(6): 947-954.

48. Lian, K., L. Wang, D. Lin and Z. Chen (2013). "Minimally invasive plating osteosynthesis for mid-distal third humeral shaft fractures." Orthopedics 36(8): e1025-1032.

49. Litchfield, R. B., M. D. McKee, R. Balyk, S. Mandel, R. Holtby, R. Hollinshead, D. Drosdowech, S. E. Wambolt, S. H. Griffin and R. McCormack (2011). "Cemented versus uncemented fixation of humeral components in total shoulder arthroplasty for osteoarthritis of the shoulder: a prospective, randomized, double-blind clinical trial-A JOINTs Canada Project." J Shoulder Elbow Surg 20(4): 529-536.

50. Liu, Q. H., Z. G. Fu, J. L. Zhou, T. Lu, T. Liu, L. Shan, Y. Liu and L. Bai (2012). "Randomized prospective study of olecranon fracture fixation: cable pin system versus tension band wiring." J Int Med Res 40(3): 1055-1066.

51. Ludvigsen, T. C., S. Johansen and S. Svenningsen (1996). "[Unstable fractures of the distal radius. External fixation or percutaneous pinning?]." Tidsskr Nor Laegeforen 116(26): 3093-3097.

52. Ludvigsen, T. C., S. Johansen, S. Svenningsen and R. Saetermo (1997). "External fixation versus percutaneous pinning for unstable Colles' fracture. Equal outcome in a randomized study of 60 patients." Acta Orthop Scand 68(3): 255-258.

53. McCormack, R. G., D. Brien, R. E. Buckley, M. D. McKee, J. Powell and E. H. Schemitsch (2000). "Fixation of fractures of the shaft of the humerus by dynamic compression plate or intramedullary nail. A prospective, randomised trial." J Bone Joint Surg Br 82(3): 336-339.

54. Mittal, R., J. Morley, H. Dinopoulos, E. G. Drakoulakis, E. Vermani and P. V. Giannoudis (2005). "Use of bio-resorbable implants for stabilisation of distal radius fractures: the United Kingdom patients' perspective." Injury 36(2): 333-338.

55. Monument, M., G. Fick and R. Buckley (2009) "Quantifying the amount of padding improves the comfort and function of a fibreglass below-elbow cast." Injury 40, 257-261 DOI: 10.1016/j.injury.2008.06.035.

56. Ockert, B., V. Pedersen, L. Geyer, S. Wirth, W. Mutschler and S. Grote (2014). "Position of polyaxial versus monoaxial screws in locked plating for proximal humeral fractures: analysis of a prospective randomized study." Eur J Orthop Surg Traumatol 24(5): 747-752.

57. O'Connor, D., H. Mullett, M. Doyle, A. Mofidi, S. Kutty and M. O'Sullivan (2003). "Minimally displaced Colles' fractures: a prospective randomized trial of treatment with a wrist splint or a plaster cast." J Hand Surg Br 28(1): 50-53.

58. Paschos, N. K., G. I. Mitsionis, H. S. Vasiliadis and A. D. Georgoulis (2013). "Comparison of early mobilization protocols in radial head fractures." J Orthop Trauma 27(3): 134-139.

59. Pieske, O., P. Geleng, J. Zaspel and S. Piltz (2008). "Titanium alloy pins versus stainless steel pins in external fixation at the wrist: a randomized prospective study." J Trauma 64(5): 1275-1280.

60. Pieske, O., F. Kaltenhauser, L. Pichlmaier, N. Schramm, H. Trentzsch, T. Loffler, A. Greiner and S. Piltz (2010). "Clinical benefit of hydroxyapatite-coated pins compared with stainless steel pins in external fixation at the wrist: a randomised prospective study." Injury 41(10): 1031-1036.

61. Pieske, O., L. Pichlmaier, F. Kaltenhauser, N. Schramm, B. Rubenbauer, A. Greiner and S. Piltz (2011). "Hydroxyapatite-coated pins versus titanium alloy pins in external fixation at the wrist: a controlled cohort study." J Trauma 70(4): 845-851.

62. Putti, A. B., R. B. Uppin and B. B. Putti (2009). "Locked intramedullary nailing versus dynamic compression plating for humeral shaft fractures." J Orthop Surg (Hong Kong) 17(2): 139-141.

63. Rangan, A., H. Handoll, S. Brealey, L. Jefferson, A. Keding, B. C. Martin, L. Goodchild, L. H. Chuang, C. Hewitt and D. Torgerson

(2015). "Surgical vs nonsurgical treatment of adults with displaced fractures of the proximal humerus: the PROFHER randomized clinical trial." Jama 313(10): 1037-1047.

64. Ribak, S., C. E. Medina, R. Mattar, Jr., H. J. Ulson, H. J. Ulson and M. Etchebehere (2010). "Treatment of scaphoid nonunion with vascularised and nonvascularised dorsal bone grafting from the distal radius." Int Orthop 34(5): 683-688.

65. Rodriguez-Merchan, E. C. (1995). "Compression plating versus hackethal nailing in closed humeral shaft fractures failing nonoperative reduction." J Orthop Trauma 9(3): 194-197.

66. Shukla, R., R. K. Jain, N. K. Sharma and R. Kumar (2014). "External fixation versus volar locking plate for displaced intra-articular distal radius fractures: a prospective randomized comparative study of the functional outcomes." J Orthop Traumatol.

67. Singh, A. K., G. R. Arun, N. Narsaria and A. Srivastava (2014). "Treatment of non-union of humerus diaphyseal fractures: a prospective study comparing interlocking nail and locking compression plate." Arch Orthop Trauma Surg 134(7): 947-953.

68. Sommerkamp, T. G., M. Seeman, J. Silliman, A. Jones, S. Patterson, J. Walker, M. Semmler, R. Browne and M. Ezaki (1994). "Dynamic external fixation of unstable fractures of the distal part of the radius. A prospective, randomized comparison with static external fixation." J Bone Joint Surg Am 76(8): 1149-1161.

69. Stoffelen, D. and P. Broos (1998). "Minimally displaced distal radius fractures: do they need plaster treatment?" J Trauma 44(3): 503-505.

70. ur Rahman, O., M. Q. Khan, H. Rasheed and S. Ahmad (2012). "Treatment of unstable intraarticular fracture of distal radius: POP casting with external fixation." J Pak Med Assoc 62(4): 358-362.

71. Uschok, S., P. Magosch, M. Moe, S. Lichtenberg and P. Habermeyer (2016). "Is the stemless humeral head replacement clinically and radiographically a secure equivalent to standard stem humeral head replacement in the long-term follow-up? A prospective randomized trial." J Shoulder Elbow Surg.

72. Wali, M. G., A. N. Baba, I. A. Latoo, N. A. Bhat, O. K. Baba and S. Sharma (2014). "Internal fixation of shaft humerus fractures by dynamic compression plate or interlocking intramedullary nail: a prospective, randomised study." Strategies Trauma Limb Reconstr 9(3): 133-140.

73. Van Leemput, T. and G. Mahieu (2007). "Conservative management of minimally displaced isolated fractures of the ulnar shaft." Acta Orthop Belg 73(6): 710-713.

74. Wang, C., G. Dai, S. Wang, Q. Liu and W. Liu (2013). "The function and muscle strength recovery of shoulder after humeral diaphysis fracture following plating and intramedullary nailing." Arch Orthop Trauma Surg 133(8): 1089-1094.

75. Varitimidis, S. E., G. K. Basdekis, Z. H. Dailiana, M. E. Hantes, K. Bargiotas and K. Malizos (2008). "Treatment of intra-articular fractures of the distal radius: fluoroscopic or arthroscopic reduction?" J Bone Joint Surg Br 90(6): 778-785.

76. Wik, T. S., A. T. Aurstad and V. Finsen (2009). "Colles' fracture: dorsal splint or complete cast during the first 10 days?" Injury 40(4): 400-404.

77. Williksen, J. H., F. Frihagen, J. C. Hellund, H. D. Kvernmo and T. Husby (2013). "Volar locking plates versus external fixation and adjuvant pin fixation in unstable distal radius fractures: a randomized, controlled study." J Hand Surg Am 38(8): 1469-1476.

78. Xu, G. G., S. P. Chan, M. E. Puhaindran and W. Y. Chew (2009). "Prospective randomised study of intra-articular fractures of the distal radius: comparison between external fixation and plate fixation." Ann Acad Med Singapore 38(7): 600-606.

79. Yuan, P., D. Liu, Q. Zan, Y. Hao, Q. Dou, G. Zhang and H. Yuan (2012) "Clinical effect of sidong wubu method in treatment of closed fracture of upper limbs." Journal of traditional Chinese medicine / Chung i tsa chih ying wen pan 32, 393-396.

80. Zaino, C. J., M. R. Patel, M. S. Arief and R. Pivec (2015). "The effectiveness of bivalving, cast spreading, and webril cutting to

reduce cast pressure in a fiberglass short arm cast." J Bone Joint Surg Am 97(5): 374-380.

Non-randomized control trials (44 trials)

1. Abbaszadegan, H., P. Adolphson, N. Dalen, U. Jonsson, H. E. Sjoberg, S. Kalen (1991). "Bone mineral loss after Colles' fracture. Plaster cast and external fixation equivalent." Acta Orthopaedica Scandinavica 62(2): 156-158.

2. Abdelhady, A. M. (2013). "Timing of shoulder arthroplasty in comminuted proximal humerus fracture, how much does it matter?" Eur J Orthop Surg Traumatol 23(5): 515-519.

3. Aktekin, C. N., M. Altay, Z. Gursoy, L. A. Aktekin, A. M. Ozturk and A. Y. Tabak (2010). "Comparison between external fixation and cast treatment in the management of distal radius fractures in patients aged 65 years and older." J Hand Surg Am 35(5): 736-742.

4. Andersen, J. R., C. D. Williams, R. Cain, M. Mighell and M. Frankle (2013). "Surgically treated humeral shaft fractures following shoulder arthroplasty." J Bone Joint Surg Am 95(1): 9-18.

5. Blonna, D., F. Castoldi, M. Scelsi, R. Rossi, G. Falcone and M. Assom (2010). "The hybrid technique: potential reduction in complications related to pins mobilization in the treatment of proximal humeral fractures." J Shoulder Elbow Surg 19(8): 1218-1229.

6. Blonna, D., R. Rossi, G. Fantino, A. Maiello, M. Assom and F. Castoldi (2009). "The impacted varus (A2.2) proximal humeral fracture in elderly patients: is minimal fixation justified? A case control study." J Shoulder Elbow Surg 18(4): 545-552.

7. Bockmann, B., B. Buecking, D. A. Eschbach, D. Franz, S. Ruchholtz and J. Mohr (2015). "Fixation of the greater tuberosity in proximal humeral fractures: FiberWire(R) or wire cerclage?" Acta Orthop Belg 81(1): 9-16.

8. Brogren, E., M. Hofer, M. Petranek, P. Wagner, L. B. Dahlin and I. Atroshi (2011). "Relationship between distal radius fracture

malunion and arm-related disability: a prospective population-based cohort study with 1-year follow-up." BMC Musculoskelet Disord 12: 9.

9. Caforio, M., P. Maniscalco, M. Colombo and G. M. Calori (2016). "Long endomedullary nail in proximal third humeral shaft fractures." Injury 47 Suppl 4: S64-s70.

10. Cai, L., S. Zhu, S. Du, W. Lin, T. Wang, D. Lu and H. Chen (2015). "The relationship between radiographic parameters and clinical outcome of distal radius fractures in elderly patients." Orthop Traumatol Surg Res 101(7): 827-831.

11. Chou, Y. C., I. C. Tseng, C. W. Chiang and C. C. Wu (2013). "Shoulder hemiarthroplasty for proximal humeral fractures: comparisons between the deltopectoral and anterolateral deltoid-splitting approaches." J Shoulder Elbow Surg 22(8): e1-7.

12. Christensen, O. M., T. C. Christiansen, M. Krasheninnikoff, B. Lind, L. R. Holmich, F. F. Hansen, G. S. Rathje and P. Holmich (2001). "Plaster cast compared with bridging external fixation for distal radius fractures of the Colles' type." Int Orthop 24(6): 358-360.

13. de Kruijf, M., J. P. Vroemen, K. de Leur, E. A. van der Voort, D. I. Vos and L. Van der Laan (2014). "Proximal fractures of the humerus in patients older than 75 years of age: should we consider operative treatment?" J Orthop Traumatol 15(2): 111-115.

14. DelSole, E. M., C. A. Pean, N. C. Tejwani and K. A. Egol (2016). "Outcome after olecranon fracture repair: Does construct type matter?" Eur J Orthop Surg Traumatol 26(2): 153-159.

15. Faraj, D., B. W. Kooistra, W. A. Vd Stappen and A. J. Werre (2011). "Results of 131 consecutive operated patients with a displaced proximal humerus fracture: an analysis with more than two years follow-up." Eur J Orthop Surg Traumatol 21(1): 7-12.

16. Hayes, A. J. H., 2008 #1523}, P. J. Duffy and M. M. McQueen (2008). "Bridging and non-bridging external fixation in the treatment of unstable fractures of the distal radius: a retrospective study of 588 patients." Acta Orthop 79(4): 540-547.

17. Hede, J. S., B. E. Lindblad, S. S. Mikkelsen and H. M. Knudsen (2000). "Comparison of intramedullary fixation and percutaneous pinning of displaced and comminuted Colles' fractures: a prospective and consecutive study." Scand J Plast Reconstr Surg Hand Surg 34(2): 161-166.

18. Hoffmann, M., M. Schroeder, K. Kossow, M. Gruber-Rathmann and A. H. Ruecker (2012). "Radiological dorsal tilt analysis of AO type A, B, and C fractures of the distal radius treated conservatively or with extra-focal K-wire plus external fixateur." Skeletal Radiol 41(9): 1133-1139.

19. Ilchmann, T., P. E. Ochsner, H. Wingstrand and K. Jonsson (1998). "Non-operative treatment versus tension-band osteosynthesis in three- and four-part proximal humeral fractures. A retrospective study of 34 fractures from two different trauma centers." Int Orthop 22(5): 316-320.

20. Jung, W. B., E. S. Moon, S. K. Kim, D. Kovacevic and M. S. Kim (2013). "Does medial support decrease major complications of unstable proximal humerus fractures treated with locking plate?" BMC Musculoskelet Disord 14: 102.

21. King, J. J., K. W. Farmer, A. M. Struk, T. W. Wright (2014). "Uncemented versus cemented humeral stem fixation in reverse shoulder arthroplasty." International Orthopaedics. 39(2):291-8.

22. Kurowicki, J., J. J. Triplet, E. Momoh, M. A. Moor and J. C. Levy (2016). "Reverse shoulder prosthesis in the treatment of locked anterior shoulders: a comparison with classic reverse shoulder indications." J Shoulder Elbow Surg 25(12): 1954-1960.

23. Larouche, J., J. Pike, G. Slobogean, P. Guy, H. Broekhuyse, P. O'Brien and K. Lefaivre (2016). "Determinants of Functional Outcome in Distal Radius Fractures in High-Functioning Patients over the age of 55." J Orthop Trauma.

24. Lee, J. I., J. H. Cho and S. J. Lee (2015). "The effects of the Frag-Loc((R)) compression screw on distal radius fracture with a displaced dorsoulnar fragment." Arch Orthop Trauma Surg 135(9): 1315-1321.

25. Lovy, A. J., A. Keswani, S. M. Koehler, J. Kim, M. Hausman (2016). "Short-Term Complications of Distal Humerus Fractures in Elderly Patients: Open Reduction Internal Fixation Versus Total Elbow Arthroplasty." Geriatric Orthopaedic Surgery and Rehabilitation 7(1): 39-44.

26. Milin, L., F. Sirveaux, F. Eloy, D. Mainard, D. Mole and H. Coudane (2014). "Comparison of modified Hackethal bundle nailing versus anterograde nailing for fixation of surgical neck fractures of the humerus: retrospective study of 105 cases." Orthop Traumatol Surg Res 100(3): 265-270.

27. Neuhaus, V., A. G. Bot, C. H. Swellengrebel, N. B. Jain, J. J. Warner and D. C. Ring (2014). "Treatment choice affects inpatient adverse events and mortality in older aged inpatients with an isolated fracture of the proximal humerus." J Shoulder Elbow Surg 23(6): 800-806.

28. Ockert, B., F. Haasters, G. Siebenbürger, T. Helfen, M. Daferner and W. Böcker (2016). "Complications of locked plating for proximal humeral fractures–are we getting any better?" Journal of Shoulder and Elbow Surgery 25(10): e295-e303.

29. Ong, C., C. Bechtel, M. Walsh, J. D. Zuckerman and K. A. Egol (2011). "Three- and four-part fractures have poorer function than one-part proximal humerus fractures." Clin Orthop Relat Res 469(12): 3292-3299.

30. Radulescu, R., A. Badila, O. Nutiu, I. Japie, S. Terinte, D. Radulescu and R. Manolescu (2014). "Osteosynthesis in fractures of the distal third of humeral diaphysis." Maedica (Buchar) 9(1): 44-48.

31. Rhee, Y. G., N. S. Cho and S. C. Moon (2015). "Effects of humeral component retroversion on functional outcomes in reverse total shoulder arthroplasty for cuff tear arthropathy." J Shoulder Elbow Surg 24(10): 1574-1581.

32. Roderer, G., J. Erhardt, M. Kuster, P. Vegt, C. Bahrs, L. Kinzl and F. Gebhard (2011). "Second generation locked plating of proximal humerus fractures--a prospective multicentre observational study." Int Orthop 35(3): 425-432.

33. Schairer, W. W., B. U. Nwachukwu, S. Lyman, E. V. Craig and L. V. Gulotta (2015). "Reverse shoulder arthroplasty versus hemiarthroplasty for treatment of proximal humerus fractures." J Shoulder Elbow Surg 24(10): 1560-1566.

34. Sirveaux, F., D. Saragaglia, D. Block, F. Gadea, R. Bouchet, T. D'ollonne, J. Gaillot and S. the (2016). "Non-operative treatment of four-part fractures of the proximal end of the humerus: results of a prospective and retrospective multicentric study." International Orthopaedics 40(8): 1669-1674.

35. Slobogean, G. P., P. Guy, J. Pike, P. O'Brien, K. A. Lefaivre, H. Broekhuyse and J. Larouche (2016). "Determinants of Functional Outcome in Distal Radius Fractures in High-Functioning Patients Older Than 55 Years." J Orthop Trauma 30(8): 445-449.

36. Smith, A. M., R. M. Mardones, J. W. Sperling and R. H. Cofield (2007). "Early complications of operatively treated proximal humeral fractures." J Shoulder Elbow Surg 16(1): 14-24.

37. Smucny, M., M. E. Menendez, D. Ring, B. T. Feeley and A. L. Zhang (2015). "Inpatient surgical site infection after shoulder arthroplasty." J Shoulder Elbow Surg 24(5): 747-753.

38. Solberg, B. D., C. N. Moon, D. P. Franco and G. D. Paiement (2009). "Surgical treatment of three and four-part proximal humeral fractures." J Bone Joint Surg Am 91(7): 1689-1697.

39. Solgaard, S., C. Bunger and K. Sllund (1990). "Displaced distal radius fractures. A comparative study of early results following external fixation, functional bracing in supination, or dorsal plaster immobilization." Arch Orthop Trauma Surg 109(1): 34-38.

40. Sudkamp, N. P., L. Audige, S. Lambert, R. Hertel and G. Konrad (2011). "Path analysis of factors for functional outcome at one year in 463 proximal humeral fractures." J Shoulder Elbow Surg 20(8): 1207-1216.

41. Tan, V., W. Bratchenko, A. Nourbakhsh and J. Capo (2012). "Comparative analysis of intramedullary nail fixation versus casting for treatment of distal radius fractures." J Hand Surg Am 37(3): 460-468.

42. Thorsness, R., J. Iannuzzi, K. Noyes, S. Kates and I. Voloshin (2014). "Open reduction and internal fixation versus hemiarthroplasty in the management of proximal humerus fractures." Geriatr Orthop Surg Rehabil 5(2): 56-62.

43. Ueda, K., S. Ikemura, A. Yamashita, T. Harada, T. Watanabe, K. Shirasawa (2014). "Three-dimensional analyses of proximal humeral fractures using computed tomography with multiplanar reconstruction: early stability of fixation after osteosynthesis in relation to preoperative bone quality." European Journal of Orthopaedic Surgery and Traumatology 24(8): 1389-1394.

44. Wang, B., K. Chen, J. Fan, B. Jiang, G. R. Yu, J. Mei and F. Yuang (2016). "Analysis of soft-tissue complications of volar plate fixation for managing distal radius fractures and clinical effect while preserving pronator quadratus." Acta Orthop Belg 82(2): 305-312.

Reasons: Studies with high risk of bias

Randomized control trials (13 trials)

1. Horne, J. G., P. Devane and G. Purdie (1990). "A prospective randomized trial of external fixation and plaster cast immobilization in the treatment of distal radial fractures." J Orthop Trauma 4(1): 30-34.

2. Ismatullah (2012). "Efficacy of plaster casting versus external fixation in comminuted distal radius fractures." Journal of Postgraduate Medical Institute 26(3): 311-316.

3. Jakubietz, R. G., J. G. Gruenert, D. F. Kloss, S. Schindele and M. G. Jakubietz (2008). "A randomised clinical study comparing palmar and dorsal fixed-angle plates for the internal fixation of AO C-type fractures of the distal radius in the elderly." J Hand Surg Eur Vol 33(5): 600-604.

4. Jeudy, J., V. Steiger, P. Boyer, P. Cronier, P. Bizot and P. Massin (2012). "Treatment of complex fractures of the distal radius: a prospective randomised comparison of external fixation 'versus' locked volar plating." Injury 43(2): 174-179.

5. Kopylov, P., K. Runnqvist, K. Jonsson and P. Aspenberg (1999). "Norian SRS versus external fixation in redisplaced distal radial fractures. A randomized study in 40 patients." Acta Orthop Scand 70(1): 1-5.

6. Koshimune, M., M. Kamano, K. Takamatsu and H. Ohashi (2005). "A randomized comparison of locking and non-locking palmar plating for unstable Colles' fractures in the elderly." J Hand Surg Br 30(5): 499-503.

7. Ledingham, W. M., R. Wytch, C. C. Goring, A. B. Mathieson and D. Wardlaw (1991). "On immediate functional bracing of Colles' fracture." Injury 22(3): 197-201.

8. Ma, C., Q. Deng, H. Pu, X. Cheng, Y. Kan, J. Yang, A. Yusufu, L. Cao (2016). "External fixation is more suitable for intra-articular fractures of the distal radius in elderly patients." Bone Research 4 Article Number: 16017.

9. Mardani Kivi, M., K. Asadi, K. Hashemi Motlagh and M. Shakiba (2011) "Distal radius fracture, a comparison between closed reduction and long arm cast Vs. Closed reduction and percutaneous pinning and short arm cast." Shiraz E Medical Journal 12, 155-161.

10. Moroni, A., F. Vannini, C. Faldini, F. Pegreffi and S. Giannini (2004). "Cast vs external fixation: a comparative study in elderly osteoporotic distal radial fracture patients." Scand J Surg 93(1): 64-67.

11. Pritchett, J. W. (1995). "External fixation or closed medullary pinning for unstable Colles fractures?" J Bone Joint Surg Br 77(2): 267-269.

12. Rajan, G. P., J. Fornaro, O. Trentz and R. Zellweger (2006). "Cancellous allograft versus autologous bone grafting for repair of comminuted distal radius fractures: a prospective, randomized trial." J Trauma 60(6): 1322-1329.

13. Saddiki, R., X. Ohl, X. Hemery, F. Vitry, E. Dehoux and A. Harisboure (2012). "Dorsally displaced distal radius fractures: comparative study of Py's and Kapandji's techniques." Orthop Traumatol Surg Res 98(1): 61-67.

Non-randomized control trials (30 trials)

1. Alm-Paulsen, P. S., O. Rod, K. Rod, B. Rajabi, H. Russwurm and V. Finsen (2012). "Percutaneous pinning of fractures of the distal radius." J Plast Surg Hand Surg 46(3-4): 195-199.

2. Arora, R., M. Gabl, M. Gschwentner, C. Deml, D. Krappinger and M. Lutz (2009). "A comparative study of clinical and radiologic outcomes of unstable colles type distal radius fractures in patients older than 70 years: nonoperative treatment versus volar locking plating." J Orthop Trauma 23(4): 237-242.

3. Bachelier, F., A. Pizanis, J. Schwitalla, T. Pohlemann, D. Kohn and R. Wirbel (2014). "Treatment for displaced proximal humerus fractures: comparison of interlocking plate fixation versus minimal invasive techniques." Eur J Orthop Surg Traumatol 24(5): 707-714.

4. Boileau, P., M. Winter, A. Cikes, Y. Han, M. Carles, G. Walch and D. G. Schwartz (2013). "Can surgeons predict what makes a good hemiarthroplasty for fracture?" J Shoulder Elbow Surg 22(11): 1495-1506.

5. Bonnevialle, N., C. Tournier, P. Clavert, X. Ohl, F. Sirveaux and D. Saragaglia (2016). "Hemiarthroplasty versus reverse shoulder arthroplasty in 4-part displaced fractures of the proximal humerus: Multicenter retrospective study." Orthop Traumatol Surg Res.

6. Carbone, S., M. Tangari, S. Gumina, R. Postacchini, A. Campi and F. Postacchini (2012). "Percutaneous pinning of three- or four-part fractures of the proximal humerus in elderly patients in poor general condition: MIROS(R) versus traditional pinning." Int Orthop 36(6): 1267-1273.

7. Chan, Y. H., T. L. Foo, C. J. Yeo and W. Y. Chew (2014). "Comparison between cast immobilization versus volar locking plate fixation of distal radius fractures in active elderly patients, the Asian perspective." Hand Surg 19(1): 19-23.

8. Gumina, S., V. Candela, P. Baudi and G. Campochiaro (2016). "Hertel 7 fracture of the humeral head. Can two different fixation

systems (Diphos/PHP) lead to different outcomes? A retrospective study." Injury 47: S59-s63.

9. Gunay, C., O. F. Oken, O. Y. Yavuz, S. H. Gunay and H. Atalar (2015). "Which modality is the best choice in distal radius fractures treated with two different Kirschner wire fixation and immobilization techniques?" Ulus Travma Acil Cerrahi Derg 21(2): 119-126.

10. Haddad, M., B. Jacoby, L. Snerum, J. Hede and S. Overgaard (2000). "External fixation of distal radial fractures: 3 or 5 weeks of external fixation." Int Orthop 24(4): 224-226.

11. Handschin, A. E., M. Cardell, C. Contaldo, O. Trentz and G. A. Wanner (2008). "Functional results of angular-stable plate fixation in displaced proximal humeral fractures." Injury 39(3): 306-313.

12. Hepp, P., J. Theopold, C. Voigt, T. Engel, C. Josten and H. Lill (2008). "The surgical approach for locking plate osteosynthesis of displaced proximal humeral fractures influences the functional outcome." J Shoulder Elbow Surg 17(1): 21-28.

13. Hung, L. P., Y. F. Leung, W. Y. Ip and Y. L. Lee (2015). "Is locking plate fixation a better option than casting for distal radius fracture in elderly people?" Hong Kong Med J 21(5): 407-410.

14. Jaura, G., J. Sikdar and S. Singh (2014). "Long Term Results of PHILOS Plating and Percutaneous K-Wire Fixation in Proximal Humerus Fractures in The Elderly." Malays Orthop J 8(1): 4-7.

15. Jawa, A., P. McCarty, J. Doornberg, M. Harris and D. Ring (2006). "Extra-articular distal-third diaphyseal fractures of the humerus. A comparison of functional bracing and plate fixation." J Bone Joint Surg Am 88(11): 2343-2347.

16. Krause, F. G., L. Huebschle and R. Hertel (2007). "Reattachment of the tuberosities with cable wires and bone graft in hemiarthroplasties done for proximal humeral fractures with cable wire and bone graft: 58 patients with a 22-month minimum follow-up." J Orthop Trauma 21(10): 682-686.

17. Krishnan, S. G., J. R. Reineck, P. D. Bennion, L. Feher and W. Z. Burkhead, Jr. (2011). "Shoulder arthroplasty for fracture: does a fracture-specific stem make a difference?" Clin Orthop Relat Res 469(12): 3317-3323.

18. Ledingham, W. M., R. Wytch, C. C. Goring, A. B. Mathieson and D. Wardlaw (1991). "On immediate functional bracing of Colles' fracture." Injury 22(3): 197-201.

19. Lee, Y. S., T. Y. Wei, Y. C. Cheng, T. L. Hsu and C. R. Huang (2012). "A comparative study of Colles' fractures in patients between fifty and seventy years of age: percutaneous K-wiring versus volar locking plating." Int Orthop 36(4): 789-794.

20. Li, F., Y. Zhu, Y. Lu, X. Liu, G. Wu and C. Jiang (2014). "Hemiarthroplasty for the treatment of complex proximal humeral fractures: does a trabecular metal prosthesis make a difference? A prospective, comparative study with a minimum 3-year follow-up." J Shoulder Elbow Surg 23(10): 1437-1443.

21. Lin, T., B. Xiao, X. Ma, D. Fu and S. Yang (2014). "Minimally invasive plate osteosynthesis with a locking compression plate is superior to open reduction and internal fixation in the management of the proximal humerus fractures." BMC Musculoskelet Disord 15: 206.

22. Liu, K., P. C. Liu, R. Liu and X. Wu (2015). "Advantage of minimally invasive lateral approach relative to conventional deltopectoral approach for treatment of proximal humerus fractures." Medical Science Monitor 21: 496-504.

23. Liu, R., P. Liu, H. Shu, J. Gong, Q. Sun, J. Wu, X. Nie, Y. Yang and M. Cai (2015). "Comparison of primary radial head replacement and ORIF (open reduction and internal fixation) in Mason type III fractures: a retrospective evaluation in 72 elderly patients." Med Sci Monit 21: 90-93.

24. Lutz, K., K. M. Yeoh, J. C. MacDermid, C. Symonette and R. Grewal (2014). "Complications associated with operative versus nonsurgical treatment of distal radius fractures in patients aged 65 years and older." J Hand Surg Am 39(7): 1280-1286.

25. Maire, N., F. Lebailly, A. Zemirline, A. Hariri, S. Facca and P. Liverneaux (2013). "Prospective continuous study comparing intrafocal cross-pinning HK2((R)) with a locking plate in distal radius fracture fixation." Chir Main 32(1): 17-24.

26. Okike, K., O. C. Lee, H. Makanji, J. H. Morgan, M. B. Harris and M. S. Vrahas (2015). "Comparison of locked plate fixation and nonoperative management for displaced proximal humerus fractures in elderly patients." Am J Orthop (Belle Mead NJ) 44(4): E106-112.

27. Robinson, C. M. and J. Christie (1993). "The two-part proximal humeral fracture: a review of operative treatment using two techniques." Injury 24(2): 123-125.

28. Solarino, G., G. Vicenti, A. Abate, M. Carrozzo, G. Picca, A. Colella and B. Moretti (2016). "Volar locking plate vs epibloc system for distal radius fractures in the elderly." Injury.

29. Synn, A. J., E. C. Makhni, M. C. Makhni, T. D. Rozental and C. S. Day (2009). "Distal radius fractures in older patients: is anatomic reduction necessary?" Clin Orthop Relat Res 467(6): 1612-1620.

30. Tamimi, I., G. Montesa, F. Collado, D. Gonzalez, P. Carnero, F. Rojas, M. Nagib, V. Perez, M. Alvarez and F. Tamimi (2015). "Displaced proximal humeral fractures: when is surgery necessary?" Injury 46(10): 1921-1929.

Studies excluded, reason: not involving humerus fractures

Randomized control trials (31 trials)

1. Abbaszadegan, H. and U. Jonsson (1990). "External fixation or plaster cast for severely displaced Colles' fractures? Prospective 1-year study of 46 patients." Acta Orthop Scand 61(6): 528-530.

2. Arora, R., M. Lutz, C. Deml, D. Krappinger, L. Haug and M. Gabl (2011). "A prospective randomized trial comparing nonoperative treatment with volar locking plate fixation for displaced and unstable distal radial fractures in patients sixty-five years of age and older." J Bone Joint Surg Am 93(23): 2146-2153.

3. Atroshi, I., E. Brogren, G. U. Larsson, J. Kloow, M. Hofer and A. M. Berggren (2006). "Wrist-bridging versus non-bridging external fixation for displaced distal radius fractures: a randomized assessor-blind clinical trial of 38 patients followed for 1 year." Acta Orthop 77(3): 445-453.

4. Azzopardi, T., S. Ehrendorfer, T. Coulton and M. Abela (2005). "Unstable extra-articular fractures of the distal radius: a prospective, randomised study of immobilisation in a cast versus supplementary percutaneous pinning." J Bone Joint Surg Br 87(6): 837-840.

5. Bartl, C., D. Stengel, T. Bruckner and F. Gebhard (2014). "The treatment of displaced intra-articular distal radius fractures in elderly patients." Dtsch Arztebl Int 111(46): 779-787.

6. Cassidy, C., J. B. Jupiter, M. Cohen, M. Delli-Santi, C. Fennell, C. Leinberry, J. Husband, A. Ladd, W. R. Seitz and B. Constanz (2003). "Norian SRS cement compared with conventional fixation in distal radial fractures. A randomized study." J Bone Joint Surg Am 85-a(11): 2127-2137.

7. Costa, M. L., J. Achten, N. R. Parsons, A. Rangan, D. Griffin, S. Tubeuf and S. E. Lamb (2014). "Percutaneous fixation with Kirschner wires versus volar locking plate fixation in adults with dorsally displaced fracture of distal radius: randomised controlled trial." Bmj 349: g4807.

8. Foldhazy, Z. and A. Leif (2010) "External fixation versus closed treatment of displaced distal radial fractures in elderly patients: A randomized controlled trial." Current orthopaedic practice 21, 288-295 DOI: 10.1097/BCO.0b013e3181cd6513.

9. Goehre, F., W. Otto, S. Schwan, T. Mendel, P. P. Vergroesen and L. Lindemann-Sperfeld (2014). "Comparison of palmar fixed-angle plate fixation with K-wire fixation of distal radius fractures (AO A2, A3, C1) in elderly patients." J Hand Surg Eur Vol 39(3): 249-257.

10. Gradl, G., G. Gradl, M. Wendt, T. Mittlmeier, G. Kundt and J. B. Jupiter (2013). "Non-bridging external fixation employing multiplanar K-wires versus volar locked plating for dorsally

displaced fractures of the distal radius." Arch Orthop Trauma Surg 133(5): 595-602.

11. Gradl, G., N. Mielsch, M. Wendt, S. Falk, T. Mittlmeier, P. Gierer and G. Gradl (2014). "Intramedullary nail versus volar plate fixation of extra-articular distal radius fractures. Two year results of a prospective randomized trial." Injury 45 Suppl 1: S3-8.

12. Hegeman, J. H., J. Oskam, J. Palen, H. J. Duis and P. A. M. Vierhout (2004) "Primary external fixation versus plaster immobilization of the intra-articular unstable distal radial fracture in the elderly." Aktuelle Traumatologie 34, 64-70 DOI: 10.1055/s-2004-815749.

13. Jakubietz, M. G., J. G. Gruenert and R. G. Jakubietz (2011). "The use of beta-tricalcium phosphate bone graft substitute in dorsally plated, comminuted distal radius fractures." J Orthop Surg Res 6: 24.

14. Jakubietz, M. G., J. G. Gruenert and R. G. Jakubietz (2012). "Palmar and dorsal fixed-angle plates in AO C-type fractures of the distal radius: is there an advantage of palmar plates in the long term?" J Orthop Surg Res 7(1): 8.

15. Kelly, A. J., D. Warwick, T. P. Crichlow and G. C. Bannister (1997) "Is manipulation of moderately displaced Colles' fracture worthwhile? A prospective randomized trial." Injury 28, 283-287.

16. Kim, J. K., Y. D. Koh and S. H. Kook (2011). "Effect of calcium phosphate bone cement augmentation on volar plate fixation of unstable distal radial fractures in the elderly." J Bone Joint Surg Am 93(7): 609-614.

17. Marcheix, P. S., A. Dotzis, P. E. Benko, J. Siegler, J. P. Arnaud and J. L. Charissoux (2010). "Extension fractures of the distal radius in patients older than 50: a prospective randomized study comparing fixation using mixed pins or a palmar fixed-angle plate." J Hand Surg Eur Vol 35(8): 646-651.

18. McFadyen, I., J. Field, P. McCann, J. Ward, S. Nicol and C. Curwen (2011). "Should unstable extra-articular distal radial fractures be treated with fixed-angle volar-locked plates or percutaneous Kirschner wires? A prospective randomised controlled trial." Injury 42(2): 162-166.

19. McQueen, M. M. (1998). "Redisplaced unstable fractures of the distal radius. A randomised, prospective study of bridging versus non-bridging external fixation." J Bone Joint Surg Br 80(4): 665-669.

20. McQueen, M. M., C. Hajducka and C. M. Court-Brown (1996). "Redisplaced unstable fractures of the distal radius: a prospective randomised comparison of four methods of treatment." J Bone Joint Surg Br 78(3): 404-409.

21. Mellstrand Navarro, C., L. Ahrengart, H. Tornqvist and S. Ponzer (2016). "Volar Locking Plate or External Fixation With Optional Addition of K-Wires for Dorsally Displaced Distal Radius Fractures: A Randomized Controlled Study." J Orthop Trauma 30(4): 217-224.

22. Millett, P. J., N. Rushton and P. J. Millet (1995) "Early mobilization in the treatment of Colles' fracture: a 3 year prospective study." Injury 26, 671-675.

23. Sanchez-Sotelo, J., L. Munuera and R. Madero (2000) "Treatment of fractures of the distal radius with a remodellable bone cement." Journal of bone and joint surgery. British volume 82, 856-863.

24. Schmalholz, A. (1990). "External skeletal fixation versus cement fixation in the treatment of redislocated Colles' fracture." Clin Orthop Relat Res(254): 236-241.

25. Schonnemann, J. O., T. B. Hansen and K. Soballe (2011). "Randomised study of non-bridging external fixation compared with intramedullary fixation of unstable distal radial fractures." J Plast Surg Hand Surg 45(4-5): 232-237.

26. Strohm, P. C., C. A. Muller, T. Boll and U. Pfister (2004). "Two procedures for Kirschner wire osteosynthesis of distal radial fractures. A randomized trial." J Bone Joint Surg Am 86-a(12): 2621-2628.

27. Tanaka, H., T. Hatta, K. Sasajima, E. Itoi and T. Aizawa (2016). "Comparative study of treatment for distal radius fractures with two different palmar locking plates." J Hand Surg Eur Vol 41(5): 536-542.

28. Tumia, N., D. Wardlaw, J. Hallett, R. Deutman, S. A. Mattsson and B. Sanden (2003). "Aberdeen Colles' fracture brace as a treatment for Colles' fracture. A multicentre, prospective, randomised, controlled trial." J Bone Joint Surg Br 85(1): 78-82.

29. Vang Hansen, F., H. Staunstrup and S. Mikkelsen (1998). "A comparison of 3 and 5 weeks immobilization for older type 1 and 2 Colles' fractures." J Hand Surg Br 23(3): 400-401.

30. Wong, T. C., Y. Chiu, W. L. Tsang, W. Y. Leung, S. K. Yam and S. H. Yeung (2010). "Casting versus percutaneous pinning for extra-articular fractures of the distal radius in an elderly Chinese population: a prospective randomised controlled trial." J Hand Surg Eur Vol 35(3): 202-208.

31. Zimmermann, R., M. Gabl, M. Lutz, P. Angermann, M. Gschwentner and S. Pechlaner (2003). "Injectable calcium phosphate bone cement Norian SRS for the treatment of intra-articular compression fractures of the distal radius in osteoporotic women." Arch Orthop Trauma Surg 123(1): 22-27.

Non-randomized control trials (10 trials)

1. Board, T., A. Kocialkowski and G. Andrew (1999). "Does Kapandji wiring help in older patients? A retrospective comparative review of displaced intra-articular distal radial fractures in patients over 55 years." Injury 30(10): 663-669.

2. Cha, S. M., H. D. Shin, K. C. Kim and E. Park (2012). "Treatment of unstable distal ulna fractures associated with distal radius fractures in patients 65 years and older." J Hand Surg Am 37(12): 2481-2487.

3. Egol, K. A., M. Walsh, S. Romo-Cardoso, S. Dorsky and N. Paksima (2010). "Distal radial fractures in the elderly: operative compared with nonoperative treatment." J Bone Joint Surg Am 92(9): 1851-1857.

4. Lattmann, T., M. Dietrich, C. Meier, M. Kilgus and A. Platz (2008). "Comparison of 2 surgical approaches for volar locking plate osteosynthesis of the distal radius." J Hand Surg Am 33(7): 1135-1143.

5. Navarro, C. M., H. J. Pettersson and A. Enocson (2015). "Complications after distal radius fracture surgery: results from a Swedish nationwide registry study." J Orthop Trauma 29(2): e36-42.

6. Neidenbach, P., L. Audige, M. Wilhelmi-Mock, B. Hanson and P. De Boer (2010). "The efficacy of closed reduction in displaced distal radius fractures." Injury 41(6): 592-598.

7. Oshige, T., A. Sakai, Y. Zenke, S. Moritani and T. Nakamura (2007). "A comparative study of clinical and radiological outcomes of dorsally angulated, unstable distal radius fractures in elderly patients: intrafocal pinning versus volar locking plating." J Hand Surg Am 32(9): 1385-1392.

8. Schmelzer-Schmied, N., P. Wieloch, A. K. Martini and W. Daecke (2009). "Comparison of external fixation, locking and non-locking palmar plating for unstable distal radius fractures in the elderly." Int Orthop 33(3): 773-778.

9. Yamashita, K., Y. Zenke, A. Sakai, T. Oshige, S. Moritani and T. Maehara (2015). "Comparison of Functional Outcome Between Early and Delayed Internal Fixation Using Volar Locking Plate for Distal Radius Fractures." J uoeh 37(2): 111-119.

10. Zenke, Y., A. Sakai, T. Oshige, S. Moritani, Y. Fuse, T. Maehara and T. Nakamura (2011). "Clinical results of volar locking plate for distal radius fractures: conventional versus minimally invasive plate osteosynthesis." J Orthop Trauma 25(7): 425-431.

Excluded publications – Health economic evaluations

Reasons: Studies not involving humerus fractures

1. Farner, S., A. Malkani, E. Lau, J. Day, J. Ochoa and K. Ong (2014). "Outcomes and cost of care for patients with distal radius fractures." Orthopedics 37(10): e866-878.

2. Koenig, K. M., G. C. Davis, M. R. Grove, A. N. A. Tosteson, K. J. Koval, A. (2009). "Is early internal fixation preferred to cast treatment for well-reduced unstable distal radial fractures?" Journal of Bone and Joint Surgery - Series A 91(9): 2086-2093.

3. Moon, C. N., C. A. Lin and S. S. Rajaee (2016). "Primary total elbow arthroplasty for distal humeral fractures in elderly patients: a nationwide analysis." Journal of Shoulder and Elbow Surgery 25(11): 1854-1860.

4. Pritchett, J. W., A. Author, U. o. Department of Orthopaedic Surgery, (1995). "External fixation or closed medullary pinning for unstable Colles fractures?" Journal of Bone and Joint Surgery - Series B 77(2): 267-269.

5. Schonnemann, J. O., T. B. Hansen and K. Soballe (2011). "Randomised study of non-bridging external fixation compared with intramedullary fixation of unstable distal radial fractures." J Plast Surg Hand Surg 45(4-5): 232-237.

6. Shauver, M. J., H. Yin, M. Banerjee, K. C. Chung, A. (2011). "Current and future national costs to medicare for the treatment of distal radius fracture in the elderly." Journal of Hand Surgery 36(8): 1282-1287.

Reasons: Studies that only studied resource use or costs, i.e. were not full health economic evaluations

1. Chalmers, P. N., W. Slikker, 3rd, N. A. Mall, A. K. Gupta, Z. Rahman, D. Enriquez and G. P. Nicholson (2014). "Reverse total shoulder arthroplasty for acute proximal humeral fracture: comparison to open reduction-internal fixation and hemiarthroplasty." J Shoulder Elbow Surg 23(2): 197-204.

2. Dean, B. J. F., L. D. Jones, A. J. R. Palmer, R. D. Macnair, P. E. Brewer, C. Jayadev, A. N. Wheelton, D. E. J. Ball, R. S. Nandra, R. S. Aujla, A. E. Sykes and A. J. Carr (2016). "A review of current surgical practice in the operative treatment of proximal humeral fractures: Does the profher trial demonstrate a need for change?" Bone and Joint Research 5(5): 178-184.

3. Handschin, A. E., M. Cardell, C. Contaldo, O. Trentz and G. A. Wanner (2008). "Functional results of angular-stable plate fixation in displaced proximal humeral fractures." Injury 39(3): 306-313.

4. Manoli, A., C. E. Capriccioso, S. R. Konda, K. A. Egol (2016). "Total shoulder arthroplasty for proximal humerus fracture is associated

with increased hospital charges despite a shorter length of stay." Orthopaedics and Traumatology: Surgery and Research 102(1): 19-24.

5. Sabharwal, S., A. W. Carter, A. Rashid, A. Darzi, P. Reilly and C. M. Gupte (2016). "Cost analysis of the surgical treatment of fractures of the proximal humerus: an evaluation of the determinants of cost and comparison of the institutional cost of treatment with the national tariff." Bone Joint J 98-b(2): 249-259.

6. Solomon, J. A., S. M. Joseph, Y. Shishani, B. N. Victoroff, J. H. Wilber, R. Gobezie and R. J. Gillespie (2016). "Cost Analysis of Hemiarthroplasty Versus Reverse Shoulder Arthroplasty for Fractures." Orthopedics: 1-6.
