# Supplementary material for: Treatment of humerus fractures in the elderly: A systematic review covering effectiveness, safety, economic aspects and evolution of practice
Source: PLoS One. 2018 Dec 13;13(12):e0207815. doi: 10.1371/journal.pone.0207815 (PMC6292626; doi:10.1371/journal.pone.0207815)
Supplement: S5 Appendix — (PDF) [file pone.0207815.s005.pdf]

S5 Appendix Cost analysis - units and unit costs

Resource utilization for treatment of proximal humerus fractures in a Swedish setting, estimated for the purpose of a HTA analysis performed by the Swedish Agency for Health Technology Assessment and Assessment of Social Services

Resources	Sling	Plate fixation	Intramedullary (IM) nail fixation	Hemiarthroplasty (HA)	Reverse shoulder arthroplasty (RSA)
Material (consumables and implants) per treatment	Sling	1 plate with associated screws, surgical dressing, sling	Nail with associated locking screw (s), surgical dressing, sling	HA, surgical dressing, sling	RSA, surgical dressing, sling
Preparation time (minutes)[1]		120	120	120	120
Operating time/ treatment time (minutes)		100 [1-7]	60[3, 8, 9]	120 [10, 11]	120 [10]
Post operation time (minutes)[1]		60	60	60	60
Orthopedic surgeon (minutes)[2]	10 [12]	120	80	140	140

Assisting orthopaedic surgeon (minutes)[3]	0	100	60	120	120
Anesthesist (minutes)[4]	0	65	65	65	65
Anaesthetic nurse (minutes) [4]	0	280	240	300	300
Surgical nurse (minutes)[4]	0	280	240	300	300
Operation assistant (minutes) [4]	0	280	240	300	300
Out-patient clinic nurse (minutes) [5]	15 [12]				
Inpatient care duration due to operation (days)[5]	0	2	2	3	3

[1] Assumption by expert senior scientists in the research group based on reported data from three large Swedish general hospitals' computerized operation planning systems.

[2] Included during operating time + 10 minutes before and 10 minutes after for preparation and documentation of surgical procedure.

[3] Included only for part of operating time. Represents time needed for training of junior orthopedic surgeons.

[4] Included during preparation time, operating time and post operation time.

[5] Assumption by expert senior scientists in the research group.

Unit costs for treatment of proximal humerus fracture in US Dollars, 2016. Estimated for the purpose of a HTA analysis performed by the Swedish Agency for Health Technology Assessment and Assessment of Social Services

Resources for proximal humerus fracture treatment	Cost per treatment	References
Sling	2	[13]
Plate fixation with screws	489	[13-15]
Intramedullary (IM) nail	489	[13-15]
Hemiarthroplasty (HA)	1363	[13-15]
Reverse shoulder arthroplasty (RSA)	3518	[13-15]
Other resources	Costs per minute	References
Theatre operating time (including operating theatre rental, overhead costs and fixed equipment)	3.2	[13]
Orthopedic surgeon	2.4	[13]
Assisting orthopedic surgeon	2.4	[13]
Anesthesist	2.4	[13]
Anaesthetic nurse	1.1	[13]
Surgical nurse	1.1	[13]
Operation assistant	0.8	[13]
Out-patient clinic nurse	0,9	[13]
Inpatient care duration due to operation (days)*	522	[13-15]

References

1. Olerud P, Ahrengart L, Ponzer S, Saving J, Tidermark J. Internal fixation versus nonoperative treatment of displaced 3-part proximal humeral fractures in elderly patients: a randomized controlled trial. Journal of shoulder and elbow surgery / American Shoulder and Elbow Surgeons [et al]. 2011;20(5):747-55. Epub 2011/03/26. doi: 10.1016/j.jse.2010.12.018. PubMed PMID: 21435907.
2. Ortmaier R, Filzmaier V, Hitzl W, Bogner R, Neubauer T, Resch H, et al. Comparison between minimally invasive, percutaneous osteosynthesis and locking plate osteosynthesis in 3-and 4-part proximal humerus fractures. BMC musculoskeletal disorders. 2015;16:297. Epub 2015/10/16. doi: 10.1186/s12891-015-0770-4. PubMed PMID: 26467533; PubMed Central PMCID: PMCPMC4607011.
3. Konrad G, Audige L, Lambert S, Hertel R, Sudkamp NP. Similar outcomes for nail versus plate fixation of three-part proximal humeral fractures. Clinical orthopaedics and related research. 2012;470(2):602-9. Epub 2011/09/01. doi: 10.1007/s11999-011-2056-y. PubMed PMID: 21879402; PubMed Central PMCID: PMCPmc3254759.
4. Handschin AE, Cardell M, Contaldo C, Trentz O, Wanner GA. Functional results of angular-stable plate fixation in displaced proximal humeral fractures. Injury. 2008;39(3):306-13. Epub 2008/02/05. doi: 10.1016/j.injury.2007.10.011. PubMed PMID: 18243202.
5. Urda A, Gonzalez A, Colino A, Lopiz Y, Garcia-Fernandez C, Marco F. Management of displaced surgical neck fractures of the humerus: health related quality of life, functional and radiographic results. Injury. 2012;43 Suppl 2:S12-9. Epub 2013/05/03. doi: 10.1016/s0020-1383(13)70174-8. PubMed PMID: 23622986.
6. Voigt C, Geisler A, Hepp P, Schulz AP, Lill H. Are polyaxially locked screws advantageous in the plate osteosynthesis of proximal humeral fractures in the elderly? A prospective randomized clinical observational study. Journal of orthopaedic trauma. 2011;25(10):596-602. Epub 2011/06/15. doi: 10.1097/BOT.0b013e318206eb46. PubMed PMID: 21670709.

7. Konrad G, Hirschmüller A, Audige L, Lambert S, Hertel R, Südkamp NP. Comparison of two different locking plates for two-, three- and four-part proximal humeral fractures - Results of an international multicentre study. International orthopaedics. 2012;36(5):1051-8. doi: 10.1007/s00264-011-1410-8.
8. Lin J. Effectiveness of locked nailing for displaced three-part proximal humeral fractures. The Journal of trauma. 2006;61(2):363-74. Epub 2006/08/19. doi: 10.1097/01.ta.0000224148.73016.30. PubMed PMID: 16917452.
9. Kazakos K, Lyras DN, Galanis V, Verettas D, Psillakis I, Chatzipappas C, et al. Internal fixation of proximal humerus fractures using the Polarus intramedullary nail. Archives of orthopaedic and trauma surgery. 2007;127(7):503-8. Epub 2007/07/11. doi: 10.1007/s00402-007-0390-z. PubMed PMID: 17619889.
10. Boyle MJ, Youn SM, Frampton CM, Ball CM. Functional outcomes of reverse shoulder arthroplasty compared with hemiarthroplasty for acute proximal humeral fractures. Journal of shoulder and elbow surgery / American Shoulder and Elbow Surgeons [et al]. 2013;22(1):32-7. Epub 2012/06/02. doi: 10.1016/j.jse.2012.03.006. PubMed PMID: 22652065.
11. Olerud P, Ahrengart L, Ponzer S, Saving J, Tidermark J. Hemiarthroplasty versus nonoperative treatment of displaced 4-part proximal humeral fractures in elderly patients: a randomized controlled trial. Journal of shoulder and elbow surgery / American Shoulder and Elbow Surgeons [et al]. 2011;20(7):1025-33. Epub 2011/07/26. doi: 10.1016/j.jse.2011.04.016. PubMed PMID: 21783385.
12. Strohm PC, Muller CA, Boll T, Pfister U. Two procedures for Kirschner wire osteosynthesis of distal radial fractures. A randomized trial. The Journal of bone and joint surgery American volume. 2004;86-a(12):2621-8. Epub 2004/12/14. PubMed PMID: 15590845.
13. Operation planning system Orbit, Södersjukhuset (Stockholm South General Hospital), Stockholm, Sweden.
14. Operation planning system Operätt, Sahlgrenska University Hospital, Gothenburg, Sweden
15. Operation planning system, Skåne University Hospital, Malmö, Sweden.
