# Supplementary material for: Treatment of humerus fractures in the elderly: A systematic review covering effectiveness, safety, economic aspects and evolution of practice
Source: PLoS One. 2018 Dec 13;13(12):e0207815. doi: 10.1371/journal.pone.0207815 (PMC6292626; doi:10.1371/journal.pone.0207815)
Supplement: S6 Appendix — (PDF) [file pone.0207815.s006.pdf]

S6 Appendix Description of studies

Description of studies included in meta-analysis regarding treatment of proximal humerus fractures, and an evaluation of the risk of bias of individual studies. All data was collected for the purpose of an HTA analysis performed by the Swedish Agency for Health Technology Assessment and Assessment of Social Services, SBU.

Proximal humerus fractures

Surgery vs non-operative treatment

**Treatment comparison**	**Studies (RCTs** and *cohort studies*)**,** *Fracture type** *Treatment*	**n**	**Age**	**Outcome measurements**	**Level of bias**	**Comments**
Hemiprosthesis vs non-operative treatment	**Boons, 2012** *Neer 4-part prox humerus fracture* *Hemiarthroplasty vs non-operative*	50	>65	Constant score<br>Complications	Low	
	**Olerud, 2011** *Neer 4-part prox humerus fracture* *Hemiarthoplasty vs non-operative*	55	>55	DASH<br>Constant score<br>EQ-5D<br>Complications	Moderate	2 years follow-up
Different internal fixations vs non-operative treatment	**Fjalestad, 2014** *Neer 3- och 4-part prox humerus fracture* *Locking plate vs non-operative*	50	>60	ASES<br>Constant score<br>15-D	Low	2 years follow-up
	**Handoll, 2015** *Neer 2-,3- och 4-part prox humerus fracture* *Surgery (ORIF (any implant) or hemiarthroplasty) vs non-operative*	250	>16<br>Mean age<br>66	Oxford Shoulder score<br>EQ-5D<br>SF-12<br>Complications	Low	2 years follow-up

	Hauschild, 2013<br>*Neer 2-part prox humerus fracture (AO-type A2 and A3)*<br>*Surgery (locking plate or nail) vs non-operative*	164	All ages.<br>Mean age 64	Constant score	Moderate/Low	133 operative vs 31 non-operative
	Innocenti, 2013<br>*Neer 2-,3- and 4-part fractures*<br>*Perkutaneous pinning vs non-operative*	51	>65	Constant score<br>VAS	Moderate/Low	
	**Olerud et al 2011**<br>*Neer 3-part prox humerus fractures*<br>*Locking plate vs non-operative*	59	>55	DASH<br>Constant score<br>EQ-5D<br>Complications	Low	2 years follow-up
	Schai, 1995<br>*Neer 3-part prox humerus fractures*<br>*Surgery (minimal intern fixation or plate or primary hemiprostesis or secondary hemiprosthesis) vs non-operative*	93	Mean age 66 (range 28–87)	Constant score	Moderate/Low	4 years follow-up<br>(range 1.5-14 years)
	**Zyto, 1997**<br>*Neer 3- och 4-part prox humerus fracture*<br>*Cerclage (Tension Band Wires) vs non-operative*	40	"Elderly patients"<br>Mean age 74	Constant score<br>ADL<br>Complications	Low	50 months follow-up

*Neer CS 2nd. Displaced proximal humeral fractures. Part I. Classification and evaluation. J Bone Joint Surg Am 1970;52:1077–89.
Brorson S, Eckardt H, Audigé L, Rolauffs B, Bahrs C. Translation between the Neer- and the AO/OTA-classification for proximal humeral fractures: do we need to be bilingual to interpret the scientific literature? BMC Res Notes 2013;6:69.

ADL: Activities of Daily living; **AO**: Arbeitsgemeintschaft für Osteosynthesefragen; **ASES**: American Shoulder and Elbow Surgeons score; **DASH**: Disabilities of the Arm Shoulder and Hand;; **EQ-5D**: EuroQoL 5 Dimensions; **ORIF**: open reduction internal fixation; **SF-12**: 12-item Short-form health survey; **VAS**: Visual Analog Scale; **15-D:** 15 dimensional HRQoL instrument

Proximal humerus fractures

Surgery vs surgery

Treatment comparison	Studies (**RCTs** and *cohort studies*), *Fracture type** *Treatment*	n	Age	Outcome measurement	Level of bias	Comments
Different types of internal fixation	**Buecking, 2014** *Neer 2-,3- och 4-part prox humerus fractures Locking plate through Deltoid split vs deltopectoral incision*	120	>18, mean age 68	Constant score ADL Complications	Low	
	Konrad, 2012 *Neer 2-,3- och 4-part prox humerus fracture LPHP plate (locking plate) vs Philos plate (locking plate)*	318	>18 Mean age 64	Constant score Complications	Moderate/Low	Prospective multicentre
	Ortmaier, 2015 *Neer 3-and 4-part proximal humerus fractures Locking plate vs Humerus block (HB)*	60	Mean age 61 (range 36–80)	Constant score	Moderate/Low	Minimum of 24 months, 36-38 months follow-up
	Shi, 2011 *Neer 2-,3- and 4-part prox humerus fractures Polyaxial vs monoaxial locking plates*	76	Mean age 69 (range 60–81)	Constant Complications	Moderate/Low	
	**Voigt, 2011** *Neer 3- and 4-part prox humerus fractures Polyaxial vs monoyaxial locking plates*	56	>60	DASH Constant score Complications	Low	

	Yan, 2012<br>*Neer 2-,3-part prox humerus fractures*<br>*T-plate vs locking plate*	91	All ages, mean age 68	ASES<br>HSS	Moderate/Low	14-45 months follow-up
Different types of ORIF with or without medial support	**Liu, 2011**<br>*Neer 2-,3- and 4-part prox humerus fractures*<br>*Locking plate with or without calcium sulfate reinforcement*	50	>60	Neer scoring system<br>Complications	Moderate	All patients with BMD below 2.5 (osteoporosis)
	Peng, 2012<br>*Neer 2-,3- and 4-part prox humerus fracture*<br>*Non-locking plate with or without bone allograft*	90	>65	Complications	Moderate/Low	
	**Zhang, 2011**<br>*Neer 2-part, 3-part and 4-part fracture Locking plate with or without medial support screw*	72	>18 years, mean age 63	Constant score<br>Complications	Low	
Different types of ORIF vs prosthesis	**Chen, 2016**<br>*Neer 4-part prox humerus fracture,*<br>*Locking plate with fibula graft vs hemiarthroplasty*	60	Mean age 66 (range 51-81)	DASH<br>Constant score<br>Complications	Low	All patients with BMD<-3,0 (osteoporosis)
	Ortmaier, 2015 Neer 3 and 4-part prox humerus fracture;<br>*Reverse shoulder arthroplasty (RSA) vs Humerus block*	50	>65	Constant score	Moderate/Low	
	Spross, 2012<br>*Neer group 6 prox humerus fractures*<br>*Locking plate vs hemiarthroplasty*	44	Mean age 75 (range 42–93)	Constant score<br>SF-36<br>Complications	Moderate/Low	

Plate vs nail	**Gracitelli, 2016**<br>*Neer 2-,3- part prox humerus fractures*<br>*Locking plate vs intramedullary nail*	72	50–85 years	DASH<br>Complications	Low	
	Gradl, 2009<br>*Neer 2-,3- and 4-part prox humerus fractures*<br>*Locking plate vs intramedullary nail*	152	All ages, mean age 63	Constant score<br>Complications	Moderate/Low	Prospective multicenter study
	Konrad, 2012<br>*Neer 3-part prox humerus fractures*<br>*Locking plate vs intramedullary nail*	211	>18 years, mean age 65	Constant score<br>Neer score<br>Complications	Moderate/Low	Prospective
	Urda, 2012<br>*Neer 2-part prox humerus fractures*<br>*Locking plate vs intramedullary nail*	50	All ages, mean age 70	Constant score<br>EQ-5D<br>Complications	Moderate/Low	Comparison of percutaneous pinning not included in analysis due to too few individuals, n=9<br>Mean follow-up 40 months

*Neer CS 2nd. Displaced proximal humeral fractures. Part I. Classification and evaluation. J Bone Joint Surg Am 1970;52:1077–89.
Brorson S, Eckardt H, Audigé L, Rolauffs B, Bahrs C. Translation between the Neer- and the AO/OTA-classification for proximal humeral fractures: do we need to be bilingual to interpret the scientific literature? BMC Res Notes 2013;6:69.

ADL: Activities of daily living**; ASES:** American Shoulder and Elbow Surgeons score; **BMD:** Bone Mineral Density**; DASH**: Disabilities of the arm, shoulder and hand score;
EQ-5D: EuroQoL 5 Dimensions; **LPHP**; Locking Proximal Humerus Plate**; HSS:** Hospital for Special Surgery score; **ORIF**: open reduction internal fixation; **PHILOS**: Proximal humeral internal locking system; **SF-36:** 36 item Short Form Health survey; **RSA**; Reverse shoulder arthroplasty;

**Treatment comparison**	**Studies (RCTs** and *cohort studies***),** *Fracture type** *Treatment*	**n**	**Age**	**Outcome measurement**	**Level of bias**	**Comments**
Reverse shoulder arthroplasty (RSA) vs hemiarthroplasty	*Boyle, 2013* *Prox humerus fractures treated with prosthesis: Reverse shoulder arthroplasty (RSA) vs hemiarthroplasty*	368	All ages, mean age 76	OSS Complications	Moderate/Low	5 years follow-up Registry study 313 HA, 55 RSA
	Cuff, 2013 *Neer 3-part with involvement of major tubercle major, 4-part fractures or fractures with joint surface injury Reverse shoulder arthroplasty (RSA) vs hemiarthroplasty*	53	"elderly". Mean age 74	ASES Simple shoulder test Complications	Moderate/Low	2 years follow-up
	Gallinet, 2009 *Neer 3- and 4-part prox humerus fracture Reverse shoulder arthroplasty (RSA) vs hemiarthroplasty*	40	Mean age 74 (range 49–95)	DASH Constant Complications	Moderate/Low	
	**Sebastia-Forcada, 2014** *Prox. humerus fractures Reverse shoulder arthroplasty (RSA) vs hemiarthroplasty*	62	>70	QuickDASH Constant score Complications	Low	49 months follow-up
Variations of fixation of tubercles	**Fialka 2008** *Neer 4-part prox humerus fractures Hemiarthroplasty with 2 different types of tubercle fixation*	40	>50	Constant score Complications	Moderate	Time point for outcome measurement not stated

	Loew, 2006<br>*Neer 3- and 4-part prox humerus fractures and fractures with split humeral head Hemiarthroplasty with 2 different types of tubercle fixation*	39	Mean age 72 (range 54–88)	Constant score	Moderate/Low	
	Dietz, 2012<br>*Neer 4-part prox humerus fractures Suture vs wire fixation of major tubercle during hemiartroplasty*	54	Mean age 73 (range 45–97)	Constant score	Moderate/Low	
	Wu, 2013<br>*Neer 4-part prox humerus fractures Hemiarthroplasty with 2 different types of tubercle fixation*	67	Median age 70 (range 62–88)	Neer scoring system	Moderate/Low	
Variations of intramedullary nails	**Lopiz, 2014**<br>*Neer 2- and 3-part prox humerus fractures Curvilinear vs straight intramedullary nail*	52	Mean age 70 (range 38–89)	Constant score<br>Complications	Low	

*Neer CS 2nd. Displaced proximal humeral fractures. Part I. Classification and evaluation. J Bone Joint Surg Am 1970;52:1077–89.
Brorson S, Eckardt H, Audigé L, Rolauffs B, Bahrs C. Translation between the Neer- and the AO/OTA-classification for proximal humeral fractures: do we need to be bilingual to interpret the scientific literature? BMC Res Notes 2013;6:69.

ASES: American Shoulder and Elbow Surgeons score; **DASH**: Disabilities of the Arm Shoulder and Hand**; OSS**: Oxford shoulder score; **Quick-DASH:** Quick Disabilities of the Arm Shoulder and Hand**; RSA**; Reverse shoulder arthroplasty

Distal humerus fractures

**Treatment comparison**	**Studies (RCTs** and *cohort studies*)**,** *Fracture type* Treatment*	**n**	**Age**	**Outcome measurement**	**Level of bias**	**Comments**
Variations of plates vs prosthesis	**McKee, 2009** *Distal humerus fractures AO type C ORIF (plate and screws) vs total elbow replacement*	40	>65	DASH MEPS Complications	Low	
Variations of total elbow replacements	*Prasad, 2008 Total elbow joint replacement due to distal humerus fractures, AO type A3, B3, C3 Acute vs secondary joint replacement*	32	Mean age 78 (range 61–89)	MEPS Complications	Moderate/Low	

*Müller ME, Nazarian S, Koch P, 1990, The Comprehensive Classification of Fractures of Long Bones, Berlin, Heidelberg, Springer Verlag.

AO: Arbeitsgemeintschaft für Osteosynthesefragen**; DASH**: Disabilities of the arm, shoulder and hand score; **MEPS:** Mayo Elbow Performance Score; **ORIF**: open reduction internal fixation

Diaphyseal humerus fractures

**Treatment comparison**	**Studies (RCTs** and *cohort studies*)**,** *Fracture type* *Treatment*	**n**	**Age**	**Outcome measurement**	**Level of bias**	**Comments**
Plate vs nail	***Chen, F., 2013*** *Diafyseal humerus fractures* *ORIF vs Intramedullary nail*	1385	All ages, mean age 74	Complications	Moderate/Low	Registry study

ORIF: open reduction internal fixation

Proximal humerus fractures

Rehabilitation after shoulder hemiarthroplasty

**Treatment comparison**	**Studies (RCTs** and *cohort studies*)**,** *Fracture type** *Treatment*	**n**	**Age**	**Outcome measurement**	**Level of bias**	**Comments**
Comparison of different rehab regimes	**Agorastides, 2007** *Neer 3- and 4-part prox humerus fractures* *Mobilization 2 vs 6 weeks after hemiarthroplasty*	59	"Physiologically old" Mean age 70	Oxford score Constant score Complications	Low	

*Neer CS 2nd. Displaced proximal humeral fractures. Part I. Classification and evaluation. J Bone Joint Surg Am 1970;52:1077–89.
Brorson S, Eckardt H, Audigé L, Rolauffs B, Bahrs C. Translation between the Neer- and the AO/OTA-classification for proximal humeral fractures: do we need to be bilingual to interpret the scientific literature? BMC Res Notes 2013;6:69.
